# Multiple Fra-1-bound enhancers showing different molecular and functional features can cooperate to repress gene transcription

**DOI:** 10.1186/s13578-023-01077-5

**Published:** 2023-07-18

**Authors:** Fabienne Bejjani, Emilie Evanno, Samantha Mahfoud, Claire Tolza, Kazem Zibara, Marc Piechaczyk, Isabelle Jariel-Encontre

**Affiliations:** 1 IGMM, Univ Montpellier, CNRS, Montpellier, France; 2 DSST, ER045, PRASE, Lebanese University, Beirut, Lebanon; 3 Biology Department, Faculty of Sciences-I, Lebanese University, Beirut, Lebanon; 4 Present Address: Institut de Recherche en Cancérologie de Montpellier, IRCM, INSERM U1194, ICM, Université de Montpellier, Montpellier, France

**Keywords:** AP-1, Fra-1, FOSL1, Transcription, Transcription repression, Enhancer, p300, CBP, Triple negative breast cancer

## Abstract

**Background:**

How transcription factors (TFs) down-regulate gene expression remains ill-understood, especially when they bind to multiple enhancers contacting the same gene promoter. In particular, it is not known whether they exert similar or significantly different molecular effects at these enhancers.

**Results:**

To address this issue, we used a particularly well-suited study model consisting of the down-regulation of the *TGFB2* gene by the TF Fra-1 in Fra-1-overexpressing cancer cells, as Fra-1 binds to multiple enhancers interacting with the *TGFB2* promoter. We show that Fra-1 does not repress *TGFB2* transcription via reducing RNA Pol II recruitment at the gene promoter but by decreasing the formation of its transcription-initiating form. This is associated with complex long-range chromatin interactions implicating multiple molecularly and functionally heterogeneous Fra-1-bound transcriptional enhancers distal to the *TGFB2* transcriptional start site. In particular, the latter display differential requirements upon the presence and the activity of the lysine acetyltransferase p300/CBP. Furthermore, the final transcriptional output of the *TGFB2* gene seems to depend on a balance between the positive and negative effects of Fra-1 at these enhancers.

**Conclusion:**

Our work unveils complex molecular mechanisms underlying the repressive actions of Fra-1 on *TGFB2* gene expression. This has consequences for our general understanding of the functioning of the ubiquitous transcriptional complex AP-1, of which Fra-1 is the most documented component for prooncogenic activities. In addition, it raises the general question of the heterogeneity of the molecular functions of TFs binding to different enhancers regulating the same gene.

**Supplementary Information:**

The online version contains supplementary material available at 10.1186/s13578-023-01077-5.

## Background

Gene up-regulation by transcription factors (TFs) has amply been studied. In contrast, how TFs down-regulate gene expression remains largely ill-understood. This question is all the more complex to solve that most gene promoters are now known to interact with multiple enhancers [1–5] and that several of these regulatory elements may potentially be bound by the same TFs. Moreover, in the latter case, whether the molecular role(s)/effect(s) of the same TF at the various enhancers it binds to are identical or significantly different remains an open question. As an approach to tackle this multifaceted issue in a context where gene regulation through coordinated action of multiple distal regulatory elements remains understudied, we employed a particularly well-suited model system consisting of *TGFB2* gene down-regulation by the Fra-1 TF in Fra-1-overexpressing cancer cells, as Fra-1 binds to the numerous enhancers contacting the *TGFB2* gene promoter.

Fra-1 (encoded by the *FOSL1* gene) is a component of the ubiquitous dimeric transcriptional complex AP-1. The latter is predominantly made up of combinations of members of the Fos- (c-Fos, FosB, Fra-1 and Fra-2) and Jun (c-Jun, JunB and JunD) multigene families but is also contributed to a lesser extent by members of the ATF and MAF multigene families [6–8] and plays roles in virtually all cellular and organismal functions [6–8]. Fra-1 is, by far, the Fos family protein whose implication in cancer has been most documented [9–13]. It has not been found mutated in any tumor till now. Rather, it is overexpressed in many epithelial cancers where it plays important parts in tumor progression, aggressiveness, metastasis formation and/or resistance to treatments [9–13]. Mechanistically, it acts via being both a target and an essential effector of different oncogenic signaling pathways activated in these tumors [9–13]. Oncogenic activation of these pathways can, not only result in more elevated accumulation of the Fra-1 mRNA, but also in phosphorylation at multiple sites of the Fra-1 protein itself, which can increase both its stability and its transcriptional activity [13–22]. Overexpressed, hyperphosphorylated Fra-1 entails transcriptional reprogramming of cancer cells, affecting biological processes such as cell division and survival, apoptosis, cellular plasticity, epithelial-to-mesenchymal transition (EMT), motility, invasion, metastatization and chemo- or radioresistance [9–13, 23–26]. Of particular importance for the herein work, the implication of Fra-1 in tumor aggressiveness has been particularly well studied in triple-negative breast cancers (TNBC), which account for 15% of all breast cancers (BC) and are the most-deadly BC subtype.

Contrasting with the wealth of reports addressing the physio-pathological processes regulated by Fra-1, the mechanisms whereby this TF controls gene expression have little been studied thus far [12]. Yet, such a knowledge would be essential for better molecular understanding of the various functions of Fra-1, as well as for identifying therapeutic vulnerabilities allowing to counteract its deleterious effects in cancer. It is important to underline that how the other AP-1 components molecularly regulate transcription remains ill-understood as well [8]. There is however evidence that the mechanisms underlying the actions of the AP-1 constituents may largely be gene-, cell type- and physiological/pathological conditions-specific [8]. Moreover, it is unknown to which extent they can be shared by, or be specific of, the different members of the Fos and Jun families [8]. It ensues from this that many case/situation-specific studies still need to be conducted before a complete understanding of AP-1 transcriptional functioning is achieved.

Fra-1 is a bZIP TF, i.e. a TF harboring a basic domain (b) enabling binding to DNA, adjacent to a leucine zipper (ZIP) permitting dimerization with other TFs [6]. To exert its transcriptional actions, it neither homodimerizes nor heterodimerizes with the other Fos family members but must heterodimerize with other AP-1-constituting TFs to bind to so-called AP-1/TRE- or CRE DNA-binding sites [6]. Initially, Fra-1 has been reported as a transcriptional repressor [27, 28]. Later, it rather appeared as a TF with low intrinsic gene transactivation activity that could however be potentiated by phosphorylations at various sites [14, 15, 17]. Indeed, Fra-1 was rapidly shown capable of stimulating several genes important in cancer through binding to AP-1/TRE and/or CRE motifs [12] in studies where the authors took advantage of AP-1-binding sites localized relatively close (kb range) to the genes' transcriptional start sites (TSS). However, large-scale studies have more recently refined our view of Fra-1. On the one hand, transcriptomic analyses showed that, in addition to up-regulating many genes (i.e. hundreds to thousands) in cells where it is (over-) expressed, Fra-1 is also key for down-regulating as many other genes [23–26, 29–37], suggesting that it can act as both a *trans*-activator and a *trans*-repressor in the same cell context and at the same time, depending on its target gene. On the other hand, ChIP-seq experiments showed that Fra-1 is much more often associated with genetic elements located distally or very distally (i.e. several kb up to hundreds of kb) from TSSs than with gene promoters [24, 36–41]. As most of these genetic elements show features of transcriptional enhancers, this implies that chromatin 3D organization is key for Fra-1 to properly regulate its target genes. Finally, Fra-1 was shown to functionally collaborate with non-AP-1 TFs such as TEAD family members [42–44] or NF-κB [45], and a few transcriptional cofactors such as the lysine acetyl transferase p300 [36, 46, 47], the coactivators SRC-1 to -3 [44] or the RNA helicase DDX5 [41] which were reported to collaborate with Fra-1 for *tran*s-activating certain, but not all, of its target genes. The fine mechanistical consequences of these cooperations were however not studied.

To better understand the molecular mechanisms underlying Fra-1-mediated transcription regulation in TNBCs, we turned to the model cell line MDA-MB-231 [36, 47, 48] for two reasons. On the one hand, it is one of the most widely used TNBC model cell line that recapitulates many of the biological characteristics of TNBC aggressiveness, including in animal models (see ref. 38 for details). On the other hand, it offers a favorable Fos family protein landscape, as, besides overexpressing hyperphosphorylated Fra-1, MDA-MB-231 cells express neither c-Fos nor FosB and only little amounts of Fra-2 (15-fold less than Fra-1) [36, 48]. By combining transcriptomics-, ChIP-seq-, ATAC-seq- and Next Generation (NG) Capture-C data, we recently showed that Fra-1 (i) controls a vast network of up- and down-regulated genes (> 1000 genes with a fold change >  ± 1.5), in agreement with a work by others in another TNBC cell line (BT549) [24], (ii) binds principally to chromatin regions with features of active enhancers located distally on the linear scale (median distance of 50 kb) from the TSSs of the closest genes and (iii) exerts no major role in the control of long-range chromatin interactions at target gene loci [36]. Our work also indicates strong molecular heterogeneity of Fra-1-bound enhancers, as they show differential amounts of RNA Polymerase II (Pol II), lysine acetyl transferases p300/CBP and diverse histone marks in ChIP-seq experiments [36]. Moreover, it also points to long distance interactions between Fra-1-bound- and, sometimes, Fra-1-unbound enhancers most probably assembling into regulatory hubs to control the expression of Fra-1-regulated genes [36]. Taken together, the above-described observations suggest that Fra-1 regulates the expression of its target genes owing to a multiplicity of mechanisms. Indeed, this notion is experimentally supported by the fine study of the transcriptional up-regulation by Fra-1 of two TNBC aggressiveness-contributing genes, *PLAU* (encoding the urokinase plasminogen activator) [47] and *HMGA1* (encoding the architectural chromatin protein HMGA1) [48]. For example, but not exhaustively, Fra-1 facilitates the recruitment of Pol II at the TSS of *HMGA1* but not at that of *PLAU* where it, instead, promotes the formation of the transcription-elongating form of Pol II.

Thus far, the mechanisms whereby Fra-1 regulates transcription have been studied only on genes up-regulated by Fra-1 [12]. Importantly, all of them, including *PLAU* and *HMGA1*, belonged to the minor category of those for which Fra-1-bound AP-1/TRE- and/or CRE elements reside in the proximity of the genes' TSSs (i.e. a few kbs upstream or downstream) [12]). In contrast, how Fra-1 molecularly represses transcription and how multiple long-range chromatin interactions underlie transcriptional regulation by Fra-1 have been overlooked till now. Here, we have addressed this two-fold issue in TNBCs via investigating how Fra-1 down-regulates the *TGFB2* gene (encoding the TGFβ2 cytokine, a component of the TGFβ pathway playing an important part in TNBC aggressiveness [49]), as on the one hand, *TGFB2* is one of the genes most repressed by Fra-1 in MDA-MB-231 cells and, on the other hand, its promoter interacts with multiple Fra-1-bound enhancers, some of which are located up to > 1400 kb downstream of the gene’s TSS ([36] and this work). Our data reveal a complex molecular and functional heterogeneity of these enhancers. They also impact our view of AP-1, as some of its other components can also display both positive and negative transcriptional effects depending on the gene and on the context [6, 8] and, more generally, raise important questions concerning the actions of TFs repressing gene expression via binding to multiple enhancers.

## Results

### *TGFB2* is transcriptionally repressed by Fra-1

**Fig. 1:**
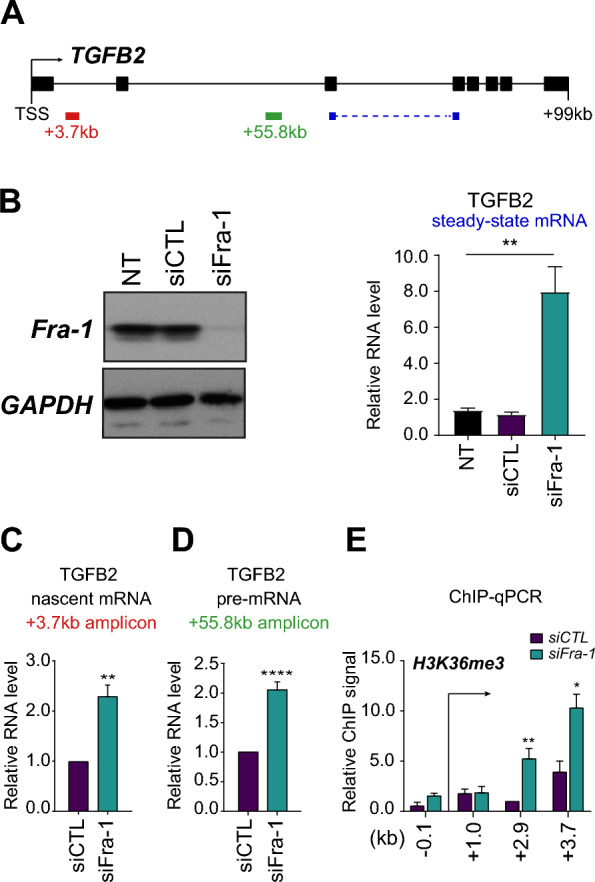
Transcriptional repression of *TGFB2* by Fra-1. In all experiments, MDA-MB-231 cells were transfected with either siCTL (control) or siFra-1 for 72 h. **A** *TGFB2 gene*. The 8 exons of *TGFB2* are indicated by black boxes. The amplicons used for assaying nascent RNAs by run-on or *TGFB2* pre-mRNA are located in the first and second introns and are indicated in red and green, respectively, whereas the one used for assaying *TGFB2* mRNA overlaps exons 3 and 4 and is shown in blue. **B** *TGFB2 mRNA abundance upon RNAi-mediated depletion of Fra-1*. Fra-1 amounts were assayed by immunoblotting, taking glyceraldehyde 3-phosphate dehydrogenase (GAPDH) as an invariant loading control (left panel). *TGFB2* mRNA was assayed by RT-qPCR in 4 independent experiments (right panel). The *S26* mRNA was taken as an internal standard and signals were normalized to the siCTL condition arbitrarily set to 1. **C** *Assay of TGFB2 nascent RNAs upon Fra-1 depletion*. Run-on assays were performed in 3 independent experiments. Quantifications were performed using *GAPDH* as an invariant standard and normalized to the siCTL condition arbitrarily set to 1. **D** *Assay of TGFB2 pre-mRNA upon Fra-1 depletion*. *TGFB2* pre-mRNA contained in total cell RNA was assayed by RT-qPCR in 7 independent experiments *GAPDH* mRNA was used as an invariant standard and data were normalized to the siCTL condition arbitrarily set to 1. **E** *Assay of H3K36me3 at the beginning of TGFB2 after Fra-1 depletion.* ChIP-qPCRs were carried out as indicated in Materials and Methods. The positions of the amplicons used are indicated with respect to the *TGFB2* TSS, which is indicated by an arrow. The values are the mean of 5 independent experiments and were normalized to that of amplicon + 2,9 under control condition, which was arbitrarily set to 1. The sequences, or the commercial references, of the siRNAs used are provided in Additional file 7: Table S1A whereas the sequences of the oligonucleotides used in RT-PCR assays are given in Additional file 7: Table S1B

The *TGFB2* gene is 99 kb-long and is made up of 8 exons (Fig. 1A). Our former Affymetrix array technology-based transcriptomic data [36] showed that *TGFB2* mRNA abundance is negatively regulated by Fra-1 in MDA-MB-231 cells and marginally, if any, by Fra-2 (see Additional file Table S1 in ref. [36]. Such a repressive role of Fra-1 on *TGFB2* mRNA was confirmed here in RT-qPCR assays showing a ≈sixfold increase in MDA-MB-231 cells transfected with a Fra-1-directed siRNA (siFra-1) as compared to cells transfected with a control siRNA (siCTL) (Fig. 1B and Additional file 1: Data S1A).

Even though our former genomic data suggested direct transcriptional repression by Fra-1 due to interactions between the *TGFB2* TSS and several Fra-1-bound candidate enhancer elements [36] (see below for more details), we decided to first formally confirm Fra-1-dependent transcriptional down-regulation of *TGFB2*. This was achieved in three ways. First, we monitored nascent RNAs produced by transcribing Pol II in run-on assays conducted in the presence and in the absence of Fra-1. RNAi-mediated Fra-1 knockdown entailed a 2- to 2.5-fold increase in transcriptional signals measured 3.7 kb downstream the *TGFB2* TSS (Fig. 1C), as well as at position + 1.5 kb (Additional file 1: Data S1B). Second, we RT-qPCR-assayed *TGFB2* pre-mRNA abundance, which showed ≈twofold higher after knockdown of Fra-1 (Fig. 1D). This was achieved using amplicons located within the second intron either at + 55.8 kb (Fig. 1A; more information on this region is provided below and in Additional file 5: Data S5) or at + 21.6 kb (Additional file 1: Data S1C). Finally, we ChIP-qPCR-assayed the abundance of H3K36me3 downstream of *TGFB2* gene transcription initiation site, as this histone mark is deposited during transcription elongation by the histone methyl transferase SETD2 that associates with the RNA-elongating form of Pol II [50]. RNAi-mediated down-regulation of Fra-1 showed a clear increase of this histone mark on the *TGFB2* gene body at positions + 2.96- and + 3.7 kb (Fig. 1E), supporting the idea of increased Pol II activity at this locus under this condition.

Thus, our data indicate that Fra-1 is key for transcriptional down-regulation of *TGFB2* in MDA-MB-231 cells. However, they also raised the possibility of a Fra-1-dependent post-transcriptional contribution to *TGFB2* gene repression, as *TGFB2* mRNA abundance was found more increased upon Fra-1 knockdown than *TGFB2* nascent RNA- or -pre-mRNA levels (see Discussion).

### Fra-1 negatively affects the activity of Pol II but not its recruitment at the *TGFB2* locus

**Fig. 2:**
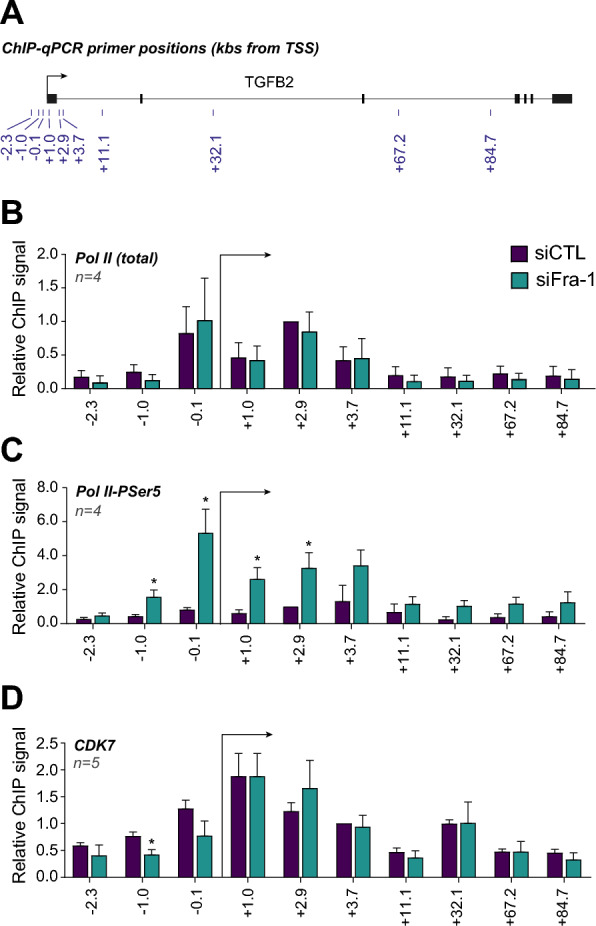
Repression of Pol II activity by Fra-1 at the *TGFB2* locus. **A** *TGFB2 gene and ChIP-qPCR amplicons*. The positions of the amplicons used in ChIP-qPCR experiments are indicated in kb from the TSS. All experiments were carried out using MDA-MB-231 cells transfected with either siFra-1 (green boxes) or siCTL (violet boxes) for 72 h. All values were normalized to that of amplicon + 2.9 kb under control condition arbitrarily set to 1. The arrows indicate the *TGFB2* TSS. **B** *ChIP-qPCR analysis of total Pol II on the TGFB2 gene*. Four independent experiments were conducted. **C** *ChIP-qPCR analysis of Pol II-PSer5 on the TGFB2 gene*. Four independent experiments were conducted. **D** *ChIP-qPCR analysis of CDK7 on the TGFB2 gene*. Five independent experiments were conducted

To explain the transcriptional action of Fra-1 on *TGFB2*, we next asked whether it could affect Pol II recruitment at this gene. This was achieved by ChIP-qPCR assay of total Pol II at various places on the *TGFB2* locus, as indicated in Fig. 2A. RNAi-mediated knockdown of Fra-1 entailed no change in Pol II abundance around the TSS and in the promoter-proximal region, as well as on the gene body (Fig. 2B). This ruled out that inhibition of Pol II recruitment was responsible for Fra-1-dependent transcriptional down-regulation of *TGFB2*. We then addressed whether Fra-1 could affect the formation of the transcription-initiating form of Pol II. The latter is phosphorylated on Serine 5 (Pol II-PSer5) of the heptad repeats of the carboxyl terminal domain (CTD) of Pol II catalytic subunit (RPB1) and is usually found enriched around TSSs and at the beginning of genes but little further on gene bodies [51]. ChIP-qPCRs conducted with an antibody specifically recognizing Pol II-PSer5 showed a significant signal increase at both the promoter and the beginning of the *TGFB2* gene after RNAi-mediated depletion of Fra-1 (Fig. 2C). As no change in Pol II and Pol II-PSer5 signals were observed on the house-keeping gene RPS26 taken as a control (Additional file 1: Data S1D), this indicated that Fra-1 negatively regulates the formation of the transcription-initiating form of Pol II at the *TGFB2* locus. As phosphorylation of RPB1 CTD Ser5 is known to be mediated by the CDK7 kinase [51], we also tested whether Fra-1 could be responsible for decreased recruitment of CDK7 at the *TGFB2* gene. Our data showed that this was not the case (Fig. 2D).

Thus, transcriptional repression of *TGFB2* by Fra-1 in MDA-MB-231 cells does not result from reduced Pol II recruitment at the *TGFB2* promoter but involves a limitation in the formation of transcription-initiating Pol II.

### The *TGFB2* promoter interacts with 15 chromatin domains localized within a single TAD

**Fig. 3:**
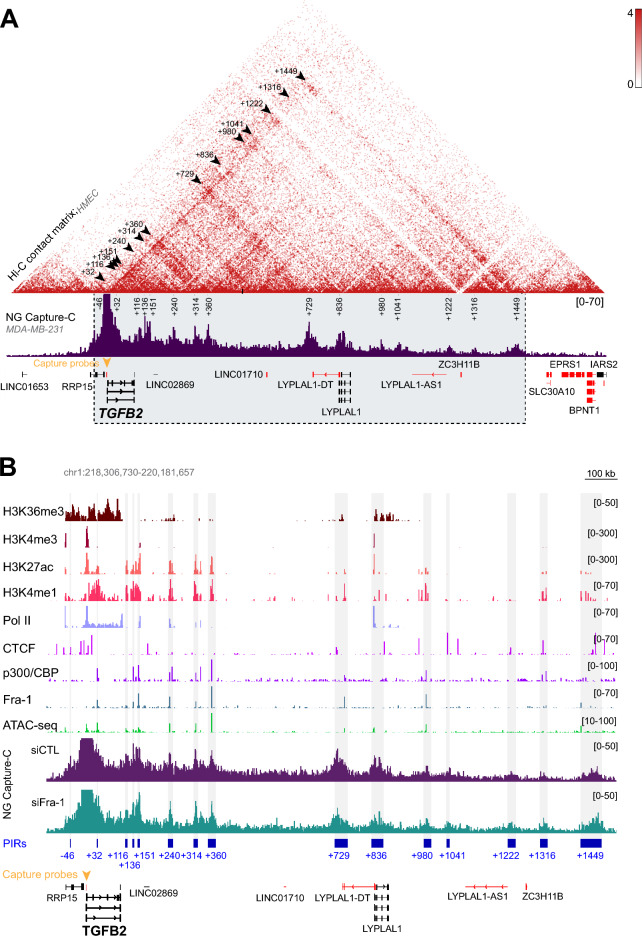
Localization and molecular characteristic of *TGFB2* PIRs within the *TGFB2* TAD. **A** *Localization of the TGFB2 PIRs within the TGFB2 TAD*. The NG Capture-C data obtained by Bejjani et al. [36] in MDA-MB-231 cells (lower panel) were aligned with the Hi-C contact matrix established by Rao et al. [55] in primary human mammary epithelial cells (HMECs) (upper panel). Normalized interaction frequency is represented as a heatmap for Hi-C data, the scale of which is shown in the upper-right corner and as a bar plot for NG-Capture C data, the scale of which is [0–70] as indicated. The yellow triangle indicates the viewpoint (*TGFB2* TSS) where the NG-Capture C probes were designed. The peaks indicate regions of interaction with the *TGFB2* promoter (PIRs). The positions of the PIR centers relative to the *TGFB2* TSS are indicated in kb. Note that the signal was strongly trimmed at the level of the viewpoint for clearer visualization of the PIRs. The genes transcribed on the forward strand are shown in black, whereas those transcribed on the reverse strand are indicated in red. The black arrows in the Hi-C data indicate the interactions identified in HMECs that correspond to the *TGFB2* PIRs identified in MDA-MB-321 cells. **B** *Molecular characterization of the TGFB2 PIRs*. NG Capture-C data obtained in the presence (siCTL condition; purple) or the absence of Fra-1 (siFra-1 condition; green) were aligned with H3K4me3-, H3K4me1-, H3K27ac-, H3K36me3, Pol II-, CTCF-, p300/CBP- and Fra-1 ChIP-seq data as well as with ATAC-seq data. The *TGFB2* PIRs are indicated by vertical grey bars, as well as by blue boxes at the bottom of the figure. Normalized read counts are indicated between brackets. All other indications are as in (**A**). NG Capture-C data were obtained in 3 independent experiments using MDA-MB-231 cells transfected with either siCTL or siFra-1 for 72 h. The 3 NG Capture-C experiments using siCTL, on the one hand, and the 3 NG Capture-C experiments using siFra-1, on the other hand, were merged, as they gave highly similar results (average Pearson correlation coefficient of 0.96)

To gain a better insight into how Fra-1 down-regulates *TGFB2* in MDA-MB-231 cells, we then further exploited our recent above-mentioned NG Capture-C results [36]. NG Capture-C is a deep sequencing-based technique allowing quantification of chromatin interactions of multiple selected viewpoints genome-wide in a single experiment and at high resolution [52]. It allowed us to identify chromatin domains interacting with the promoters of 34 selected Fra-1-up- or -down-regulated genes [36]. These domains were called Promoter-Interacting Regions or PIRs. In the gene panel studied, *TGFB2* showed 9 PIRs corresponding to peaks of sequencing reads accumulating over the background when NG Capture-C data were analyzed using the PeakC R package [53]. These PIRs were all localized downstream of the *TGFB2* gene body within the + 116 to + 980 kb region (positions + 116, + 136, + 151, + 240, + 314, + 360, + 729, + 836, + 980) (see NG Capture-C data at the bottom of Fig. 3A and B. Also see Additional file Table S4 in ref. 38). However, fine visual inspection of our NG Capture-C data coupled to the use of data from the literature, as well as of ChIP-seq data presented below, allowed us to identify additional PIRs. Four are positioned further downstream of those already identified (positions + 1041, + 1222, + 1316 and + 1449 kb). They were not identified in our initial NG Capture-C data analysis because of the stringent parameters we used to avoid selecting false positives when running the PeakC software (see Materials and Methods for details). However, we considered them here, as *TGFB2* PIR in MDA-MB-231 cells for two reasons. First, they are conserved in primary human mammary epithelial cells (HMECs), i.e. the cell type TNBCs are derived from (see Fig. 3A, upper panel). Second, all of them present features of regulatory or structural elements (see Fig. 3B and further details below). Two other candidate PIRs were also considered in the vicinity of the *TGFB2* TSS. They could not be identified by PeakC for technical reasons, as they are situated too close to the viewpoint (*TGFB2* promoter) where the sequencing background is always very high due to the intrinsic design of the NG Capture-C technology (see details on technology in reference 55). One, residing at position—46 kb, lies at the TAD border and harbors a CTCF-binding site. The other, located at position + 32 kb, corresponds to a *TGFB2* enhancer identified by the FANTOM5 consortium [54]. Moreover, it is bound by Fra-1 and displays features of an active enhancer. Interestingly, aligning our NG Capture-C data with a Hi-C contact matrix (Fig. 3A, upper panel) established in HMECs [55] showed that all PIRs (whether identified by PeakC or in our secondary analysis of NG Capture-C data), except those at − 46 and + 32 kb (which are too close to the viewpoint to be detectable), were conserved in HMECs. Moreover, all PIRs turned out to be confined within a single Topologically-Associating Domain (TAD), as defined by Rao et al. [55] (Fig. 3A). This corresponded to a classical situation, as gene regulation has principally been reported to involve intra-TAD interactions [56, 57]. Of note, one interacting region between PIRs + 360 and + 729 detected by Hi-C in HMECs [55] was not detected in the NG-Capture C experiments we conducted in MDA-MB231 cells, suggesting that the interaction between the promoter and this region is lost in the latter cells.

Interestingly, the expression of 3 other genes lying in the *TGFB2* TAD (*RRP15*, *LYPLAL1* and the long non coding gene *LINC02869*) was not affected upon RNAi-mediated knockdown of Fra-1, as assayed by Affymetrix array-based transcriptomic analysis (Additional file 1: Data S1E), pointing to the absence of Fra-1-dependent coregulation between *TGFB2* and these genes. We could however draw no conclusion for the other 4 genes residing within the *TGFB2* TAD (*ZC3H11B*, *LYPLA1-DT*, *LYPLAL1-AS*, and *LINC01710*), as they were not represented on the Affymetrix arrays we used (Additional file 1: Data S1E and Additional Table S1 in ref. 38). Nevertheless, our RNA Pol II, H3K4me3, H3K27ac and H3K36me3 ChIP-seq data (Fig. 3B) suggest that they are unlikely to be transcribed in MDA-MB-231 cells (see below for more details).

Thus, our data indicate that the *TGFB2* promoter interacts with 15 chromatin domains scattered within the same TAD and that Fra-1 does not regulate the other genes present in this TAD. As the *TGFB2* PIRs cover nearly the whole *TGFB2* TAD (Fig. 3A) and as the other genes contained in this TAD either overlap certain of these PIRs or lie between them, this suggests that a complex and/or dynamic 3D chromatin organization is at play in the *TGFB2* TAD to ensure proper expression of the genes it contains.

### The *TGFB2* PIRs are molecularly heterogeneous, suggesting functional differences

To characterize potential functional roles for *TGFB2* PIRs in *TGFB2* gene expression regulation, we next examined their individual molecular features. This was achieved via crossing our NG Capture-C data with ChIP-seq data for Fra-1, p300/CBP, several histone marks (H3K4me1, H3K4me3, H3K27ac), Pol II and CTCF, as well as with ATAC-seq data serving to identify open chromatin regions with potential regulatory roles (Fig. 3B). Thus, active promoters are usually characterized by strong H3K4me3 and H3K27ac signals, whereas active enhancers are usually marked by strong H3K4me1 and H3K27ac signals, as well as by frequent (but not mandatory) binding of p300/CBP [1–3]. On its side, Pol II is enriched at active promoters and to a much lower extent at most active enhancers [1–3], whereas CTCF is known to be involved in the structuring of chromatin interactions and is generally found in closed chromatin domains [56, 57].

Our results (Fig. 3B, Table 1 and Additional file 2: Data S2 and Additional file 3: Data S3) can be summarized as follows: (i) all PIRs bound by Fra-1 showed an AP-1/TRE site at all Fra-1 ChIP-seq peaks (Additional file 2: Data S2) strongly supporting the idea of direct binding of Fra-1 at these sites rather than that of Fra-1 being recruited by another TF through protein–protein interactions or brought about through interactions with other enhancers, (ii) PIRs + 32, + 136, + 151, + 240, + 360 and + 980 most probably bear transcriptional regulatory elements for *TGFB2* expression, as they show an open chromatin configuration, as well as features of active enhancers (low H3K4me3, high H3K4me1 and H3K27ac signals) that bind both Fra-1 and p300/CBP, (iii) PIRs − 46, + 1041, + 1222 and + 1316 most probably bear elements only involved in the 3D structuring of the *TGFB2* gene chromatin, as they show binding of CTCF but neither signs of open chromatin conformation nor of histone marks featuring active enhancers or promoters, (iv) PIR + 836, which is not bound by Fra-1, corresponds to an annotated promoter for both *LYPLAL1-ID* and *LYPLAL1* genes. It might also carry an Epromoter for *TGFB2*, i.e. a gene promoter showing enhancer activity on another gene [58], (v) PIR + 116 contains two Fra-1-bound enhancers located at + 115 kb and + 118 kb but, contrasting with the + 115 kb element showing typical marks of an active enhancer, the + 118 kb is marked by weak ATAC-seq- and p300/CBP signals, raising the possibility that the former regulatory element might be a stronger enhancer for *TGFB2* than the latter, (vi) PIR + 314 contains 2 Fra-1-bound enhancers at + 314 kb and + 315 kb, (vii) PIR + 729 contains a CTCF-bound element residing in a closed chromatin region at position + 722 kb that is likely to constitute a structural element, as well as a Fra-1- and p300/CBP-bound enhancer at + 744 kb that most probably constitutes a *TGFB2* enhancer and (viii) PIR + 1449 contains a Fra-1-bound enhancer showing a low p300/CBP signal that is located at + 1426 kb, as well as two domains binding CTCF at positions + 1460 kb and + 1468 kb that most probably correspond to chromatin-organizing elements. Finally, it must be noted that the CTCF-bound elements located within PIRs − 46 and + 1449 are located at the *TGFB2* TAD borders and might be important for the individualization of this TAD.

**Table 1:** Putative functions borne by *TGFB2* PIRs

PIR center position	PIR coordinates	Fra-1 peak	ATAC- seq	p300/CBP	CTCF	Putative function
− 46	chr1:218,471,651-218,473,869	–	–	–	− 46	Chromatin organization
+ 32	chr1:218,549,326-218,552,116	+ 32	✓	✓	–	Active enhancer
+ 116	chr1:218,630,568-218,638,881	+ 115	✓	✓	–	Active enhancer
		+ 118	✓	✓	–	Active enhancer
+ 136	chr1:218,651,100-218,656,338	+ 136	✓	✓	–	Active enhancer
+ 151	chr1:218,666,260-218,672,881	+ 151	✓	✓	–	Active enhancer
+ 240	chr1:218,753,682-218,767,687	+ 240	✓	✓	–	Active enhancer
+ 314	chr1:218,826,456-218,839,841	+ 314	✓	✓	–	Active enhancer
		+ 315	✓	✓	–	Active enhancer
+ 360	chr1:218,867,620-218,891,396	+ 360	✓	✓	–	Active enhancer
+ 729	chr1:219,232,878-219,269,584	+ 744	✓	✓		Active enhancer
		–	–	–	+ 722	Chromatin organization
+ 836	chr1:219,339,009-219,372,344	–	✓	–	–	Epromoter
+ 980	chr1:219,489,115-219,510,682	980	✓	✓	–	Active enhancer
+ 1041	chr1:219,553,878-219,563,787	–	–	–	+ 1041	Chromatin organization
+ 1222	chr1:219,730,289-219,752,737	–	–	–	+ 1222	Chromatin organization
+ 1316	chr1:219,822,398-219,844,303	–	–	+ 1314	+ 1316	Chromatin organization
+ 1449	chr1:219,939,514-220,000,504	+ 1426	✓	✓	–	Active enhancer
			–	–	+ 1460	Chromatin organization
			–	–	+ 1468	Chromatin organization

For more details, the molecular characteristics of *TGFB2* PIRs are extensively presented in Additional file 3: Data S3. Not all PIRs are bound by Fra-1, suggesting that they do not all have the same function(s). At all Fra-1-bound PIRs, Fra-1 ChIP-seq signals overlap with p300/CBP ChIP-seq- and ATAC-seq signals and are surrounded by H3K4me1 and H3K27ac ChIP-seq signals (Additional file 3: Data S3). This suggests that these PIRs bear active *TGFB2* enhancers. Besides this, certain PIRs show binding of CTCF at places neither bound by Fra-1 nor showing any detectable signals in ATAC-seq- and p300/CBP ChIP-seq experiments. As they are also poorly, or not, marked by H3K4me1, H3K4me3 and H3K27ac (Additional file 3: Data S3), they are likely to constitute 3D chromatin-organizing elements. Of note, PIRs + 729 and + 1449 are composite PIRs where a candidate Fra-1-bound active enhancer and one or two putative CTCF-bound structural elements are easily distinguished. Finally, PIR + 836 is not bound by Fra-1 but bears an annotated gene TSS and presents marks specifying active promoters, i.e. high Pol II-, H3K4me3- and H3K27ac signals, as well as ATAC-seq signal (Additional file 3: Data S3) and might be a *TGFB2* Epromoter

Thus, of the 15 *TGFB2* PIRs (see Table1), 11 appear to bear only one functional element showing features of (i) enhancers (PIR + 32, + 136, + 151, + 240, + 360, + 980), (ii) chromatin organization domains (PIR-46, + 1041, + 1222, + 1316) or (iii) a promoter possibly behaving as an Epromoter (PIR + 836). Besides this, 4 PIRs carry several functional elements. The latter can be only enhancers (PIR + 116 and + 314) or a combination of one enhancer with 1 or 2 chromatin organization domains (PIR + 729 and + 1449). The fact that the *TGFB2* promoter interacts with 12 remote Fra-1-bound elements showing marks of active enhancers (and possibly an Epromoter) strongly supports, not only the idea of *TGFB2* being a direct transcriptional target of Fra-1, but also that of a complex chromatin 3D organization/dynamics underlying *TGFB2* transcriptional regulation. This notion is strengthened by the observation that a number of CTCF-binding elements lie between certain enhancers (Table 1). Finally, it must be underlined that the various *TGFB2* enhancers (also see below) show a strong heterogeneity in Fra-1-, p300/CBP-, H3K4me1-, H3K4me3-, H3K27ac- and Pol II ChIP-seq- and ATAC-seq signal intensities (see details in Additional file 3: Data S3, which raises the notion of their functional heterogeneity.

### Fra-1 does not control long-range interactions between the *TGFB2* promoter and its cognate PIRs

We next compared NG capture-C data at the *TGFB2* locus obtained in the presence and in the absence of Fra-1 [36] to address whether Fra-1 could be instrumental in maintaining/altering enhancer-promoter interactions. Similar profiles were obtained under the two conditions (Fig. 3B, lower panels). This indicated that the negative effect of Fra-1 on *TGFB2* gene transcription is mediated by changes in neither the number nor the frequency of interactions between the gene promoter and its cognate PIRs but rather by affecting enhancer activity via other mechanisms.

### Fra-1 limits the production of eRNAs at most Fra-1-bound *TGFB2* enhancers

Active enhancers are known to be bound and bidirectionally transcribed by Pol II to generate so-called enhancer RNAs (eRNAs), the expression of which most often correlates with both enhancer activity and transcription of target genes [59–61]. To assess whether eRNAs are produced at the Fra-1-bound *TGFB2* putative enhancers, we first analyzed publicly available GRO-seq data obtained in MDA-MB-231 cells [62], as the GRO-seq technique allows visualizing both the production and the position of nascent RNA transcripts genome-wide. Short stretches of ongoing bidirectional transcription were observed at 7 of the 12 Fra-1-bound *TGFB2* PIRs (PIRs + 32, + 116, + 136, + 240, + 314, + 360 and + 1449) in the vicinity of Fra-1-binding sites (Additional file 4: Data S4). This supported the notion of eRNA synthesis, at least, at these PIRs. However, these data did not exclude that the same could also occur, but at a lower rate, at the other 5 Fra-1-bound *TGFB2* PIRs, as the GRO-seq sequencing depth might have been insufficient to detect weaker eRNA synthesis at these places. Therefore, to test whether increased transcription of *TGFB2* upon RNAi-mediated knockdown of Fra-1 could be associated to enhanced activity of all the Fra-1-bound enhancers, we turned to RT-qPCR measurement of eRNA production at these enhancers.

**Fig. 4:**
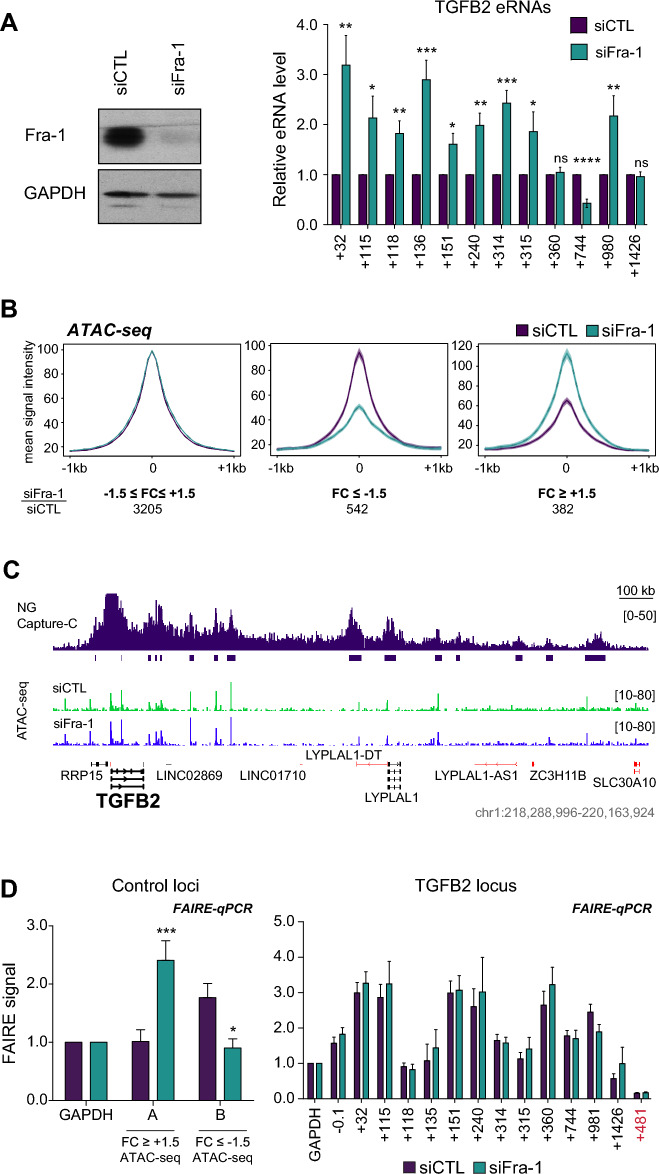
Effects of Fra-1 knockdown on the activity and accessibility of *TGFB2* enhancers. **A** *Fra-1-dependent regulation of eRNA production at most Fra-1-bound TGFB2 enhancers*. MDA-MB-231 cells were subjected to RNAi-mediated knock-down of Fra-1 for 72 h and eRNA levels were quantified by RT-qPCR at all *TGFB2* enhancers. The sequences of the PCR primers are given in Additional file 7: Table S1C. Left panel: immunoblotting analysis of Fra-1 down-regulation upon siFra-1 *versus* siCTL transfection. GAPDH was used as an invariant control. Right panel: quantifications of eRNA levels. The data correspond to 7 independent experiments using *S26* mRNA as an internal standard. They were normalized to siCTL condition arbitrarily set to 1 for each amplicon. **B** *Chromatin accessibility at all Fra-1-bound enhancers in MDA-MB-231 cells*. MDA-MB-231 cells were subjected to RNAi-mediated knockdown of Fra-1 for 72 h before ATAC-seq analyses were carried out. The metaprofiles correspond to the merge of 3 independent experiments using cells transfected with siCTL as a control. A threshold (i.e. the ratio of signal intensity in siFra-1- *versus* siCTL conditions) of ± 1.5 was used to classify the 4129 Fra-1-bound enhancers in three categories (-1.5 < FC <  + 1.5, FC ≤ − 1.5 and FC ≥ 1.5). The number of enhancers per category is indicated on the figure. **C** *Chromatin accessibility assessed by ATAC-seq in the TGFB2 TAD.* ATAC-seq- and NG Capture-C data were aligned along the whole *TGFB2* locus using the IGV browser. **D** *Chromatin accessibility assessed by FAIRE-qPCR at the Fra-1-bound TGFB2 enhancers and at the TGFB2 promoter.* The data are the mean of 8 independent experiments. The sequences of the qPCR primers are given in Additional file 7: Table S1D. Left panel: FAIRE-qPCR assay at two control positions, one shown to be more accessible (left) and one less accessible (right) upon RNAi knockdown of Fra-1 in ATAC-seq experiments conducted in MDA-MB-231 cells. Right panel: FAIRE-qPCR experiments at the *TGFB2 locus* 72 h post-transfection of siCTL or siFra-1. Position + 481 kb is devoid of any ATAC-seq signal and was used as a negative control. The *GAPDH* promoter was used as an internal control to normalize the results to compensate for differences among samples according to Rodriguez-Gil [63]

As eRNA transcription initiation site and size still remain ill-defined, we selected short amplicons (100–150 bp) located in the proximity (90–400 bp) of the Fra-1-binding sites (Additional file 7: Table S1C) to maximize the probability of eRNA detection. At variance with the other putative enhancers that are localized downstream of the *TGFB2* gene and whose transcription does not interfere with that of other transcription units, the analysis of the + 32 kb enhancer deserved a specific analysis to distinguish between production of the + 32 kb eRNAs and that of the *TGFB2* pre-mRNA due to its intronic localization. Thus, + 32 kb eRNA production was deduced from the difference in RNAs amounts between position + 32 kb and positions + 21.6 kb and + 55.8 kb, which correspond to 2 sites within intron 2 harboring no regulatory elements (lack of both ATAC-seq signals and histone modifications marking promoters or enhancers) and situated at a distance sufficient to eliminate possible overlaps between pre-mRNA- and eRNA synthesis (Additional file 5: Data S5), eRNAs being unlikely to be longer than 2–4 kb [59–61]. Our data confirmed that all putative Fra-1-bound enhancers express eRNAs and indicated that down-regulation of Fra-1 is followed by a ≈twofold enhancement of eRNA levels at 9 of the 12 enhancers (Fig. 4A). This supported the idea that (at least) these 9 enhancers are likely to control *TGFB2* expression in a Fra-1-dependent manner. Intriguingly, eRNA levels at the + 744 kb enhancer showed reproducibly diminished upon knockdown of Fra-1. This was suggestive of a positive role of Fra-1 at this specific enhancer and raised the possibility that the final *TGFB2* gene transcriptional output might depend on a balance between positive and negative transcriptional effects of Fra-1. Together with the fact that no change in eRNA levels was observed at enhancers + 360 kb and + 1426 kb upon Fra-1 down-regulation, this further argued for functional heterogeneity among the Fra-1-bound *TGFB2* enhancers.

### Fra-1 does not detectably alter chromatin accessibility at the Fra-1-bound *TGFB2* enhancers

We next asked whether increased eRNA production upon RNAi-mediated knockdown of Fra-1 could be associated with local changes in chromatin opening at Fra-1-bound *TGFB2* enhancers. To this aim, we first resorted to ATAC-seq experiments, which easily permit to assess chromatin accessibility genome-wide. In a first step, we inspected ATAC-seq signals at the level of the 4129 Fra-1-bound candidate active enhancers (cAEs) we identified in our previous work in the whole genome of MDA-MB-231 cells based on the analysis of histone modifications (see Additional file Table S2 in ref. 38). The data presented in Fig. 4B show that the siFra-1/siCTL ratio of ATAC-seq signals did not, or hardly, changed (-1.5 < fold change <  + 1.5) for the vast majority of cAEs (77.7%), decreased with a fold change ≤ -1.5 in 13.1% of the cases and increased with a fold change ≥  + 1.5 for 9.2% of them. In line with these global data, we observed no or very limited changes (i.e. <  ± 1.5-fold) in chromatin accessibility at the Fra-1-bound *TGFB2* enhancers (Fig. 4C and Additional file 6: Data S6A). To rule out the possibility of bias linked to the use of ATAC-seq, we then resorted to FAIRE-qPCR assays [63], the sensitivity of which was first tested at two arbitrarily chosen genomic positions showing either increased or decreased accessibility upon Fra-1 knockdown in ATAC-seq experiments (Fig. 4D, left panel). FAIRE-qPCR assays confirmed that Fra-1 does not affect local chromatin accessibility to any great extent at the Fra-1-bound *TGFB2* enhancers and, at the same time, also showed that the same holds true at the gene promoter (Fig. 4D, right panel). These observations are likely due to the fact that *TGFB2* transcription is only attenuated (and not strongly repressed) by Fra-1 and most probably relies on reconfiguration of the macromolecular complexes responsible for *TGFB2* expression rather than on changes in enhancer accessibility (see Discussion).

### Differential requirements upon p300/CBP binding and enzymatic activity for eRNA production at the Fra-1-bound *TGFB2* enhancers

**Fig. 5:**
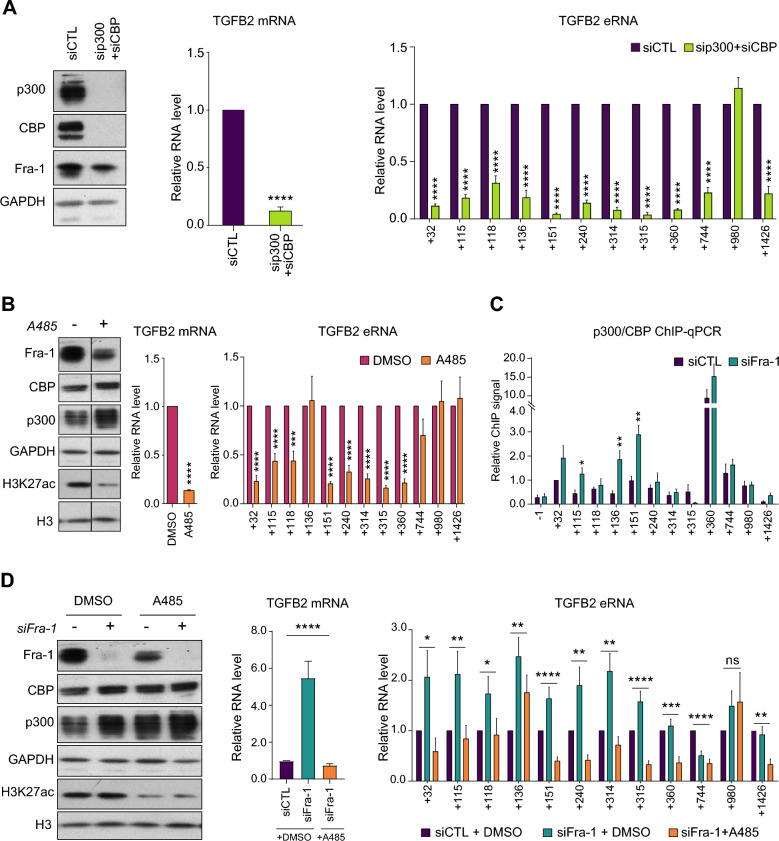
Requirement upon p300/CBP activity for eRNA expression at Fra-1-bound *TGFB2* enhancers. **A** *Dependence of p300/CBP presence for TGFB2 mRNA and eRNAs expression in MDA-MB-231 cells*. Cells were RNAi-depleted in both p300 and CBP. siCTL-transfected cells served as a reference. 72 h later, protein, mRNA and eRNA levels were analyzed. Left panel: Immunoblotting of p300 and CBP down-regulation. Fra-1 level was also assessed and GAPDH was used as an invariant control. Middle and right panels: *TGFB2* mRNA and eRNA levels, respectively. *S26* mRNA was used as an invariant internal standard. The data are the mean of 4 independent experiments in which values were normalized to that of the siCTL condition set to 1 for each amplicon. **B** *Dependence upon the activity of p300/CBP for expression of eRNAs at Fra-1-bound* TGFB2 *enhancers.* MDA-MB-231 cells were transfected with siCTL to allow comparison with the experiments shown in Fig. 5D. 56 h later, A485 or DMSO were added for 16 h and *TGFB2* mRNA or eRNAs were RT-qPCR-assayed. Left panel: immunoblotting of H3, H3K27ac, p300, CBP and Fra-1 with GAPDH used as an invariant control. Middle and right panels: *TGFB2* mRNA and eRNA levels, respectively. *S26* mRNA was used as an invariant standard. The data are the mean of 7 independent experiments in which values were normalized to the DMSO condition set to 1 for each amplicon. **C** *Dependence upon Fra-1 for recruitment of p300/CBP at the* TGFB2 *enhancers*. Cells were transfected with siCTL or siFra-1 for 72 h before p300/CBP ChIP-qPCR assays. The data are the mean of 5 independent experiments in which values were normalized to that of amplicon + 32 under control condition arbitrarily set to 1. **D** *Dependence upon the KAT activity of p300/CBP for* TGFB2 *mRNA and eRNA expression levels after RNAi-mediated depletion of Fra-1.* MDA-MB-231 cells were transfected with either siCTL or siFra-1 for 72 h, with A485 or DMSO added for the last 16 h. Left panel: immunoblotting of Fra-1, p300, CBP, histone H3 and H3K27ac, with GAPDH as an invariant control. Middle and right panels: RT-qPCR assays of *TGFB2* mRNA and eRNA levels, respectively, using *S26* mRNA as an invariant standard. The data are the mean of 7 independent experiments in which the values were normalized to that of amplicon + 32 under control condition arbitrarily set to 1

The lysine acetyl transferases p300 and CBP have long been known to be important for the activity of many, but not all, enhancers [1–3]. More recently, their enzymatic activity (KAT) was also shown to be essential for enhancer activity in many cases [64, 65]. We therefore asked whether Fra-1 could limit their recruitment and/or activity at the Fra-1-bound enhancers to repress *TGFB2* transcription. p300 and CBP are often studied together due to their close structural and functional relationships and in this case termed p300/CBP [66, 67]. We therefore depleted them together by RNAi (Fig. 5A, left panel) and RT-qPCR-assayed *TGFB2* mRNA (Fig. 5A, middle panel). Its level was reduced by 80%, indicating that p300/CBP is required for *TGFB2* gene expression. In parallel, we also tested whether p300/CBP could be instrumental for the activity of Fra-1-bound *TGFB2* enhancers by comparing eRNA levels before and after their RNAi-mediated depletion. Our data showed that 11 of the 12 enhancers require p300/CBP to be fully active (Fig. 5A, right panel). Intriguingly however, the + 980 kb enhancer displayed no change in eRNA synthesis despite a clear binding of p300/CBP (Fig. 3B, Table 1 and Additional file 3: Data S3). This nevertheless suggested that p300/CBP is not required for the activity of this specific element.

Then, we asked whether the p300/CBP KAT activity was instrumental for eRNA production at the *TGFB2* enhancers by conducting experiments in the presence of the highly specific inhibitor A485 [65, 66, 68]. We first verified A485 efficacy in siCTL-transfected MDA-MB-231 cells by showing that it strongly reduces histone H3 K27 acetylation (Fig. 5B, left panel), a histone mark specifically deposited by p300/CBP [68]. The addition of A485 also led to (i) detectable increases in overall p300 and CBP abundances, which possibly reflected induction of compensatory mechanisms in response to the drug treatment (Fig. 5B, left panel), (ii) a notable decrease in Fra-1 protein level (Fig. 5B, left panel), suggesting p300/CBP-dependent transcription of the *FOSL1* gene, and (iii) a strong reduction in *TGFB2* mRNA level, indicating that p300/CBP KAT activity is required for *TGFB2* expression (Fig. 5B, middle panel). eRNA steady-state levels, analyzed on siCTL-transfected MDA-MB-231 cells, also decreased dramatically at 8 of the 12 Fra-1-bound enhancers (+ 32 kb, + 115 kb, + 118 kb, + 151 kb, + 240 kb, + 314 kb, + 315 kb and + 360 kb) in the presence of A485 (Fig. 5B, right panel), indicating that eRNA production at these sites is dependent on p300/CBP enzymatic activity. However, no change was observed at 3 enhancers (+ 136 kb, + 744 kb and + 1426 kb), which suggested a structural/organizational, rather than an enzymatic, role for p300/CBP for production of eRNAs at these sites. Consistently with the absence of effects of p300/CBP RNAi-mediated depletion on eRNA production at enhancer + 980 kb, no change was detected at this site upon treatment with A485 either.

Thus, our data show that p300/CBP and its associated enzymatic activity are necessary for *TGFB2* gene expression in MDA-MB-231 cells in the presence of Fra-1. Moreover, they also strongly highlight the heterogeneity of the Fra-1-bound *TGFB2* enhancers with respect to their dependence on p300/CBP presence and KAT activity for eRNA production (Table 2). Thus, the activity of one of the enhancers is independent on the presence of p300/CBP even though p300/CBP binds to it and, among the 11 p300/CBP-dependent enhancers, only 8 of them require the KAT activity. This indirectly suggests that p300/CBP plays a structural/organizational role at the other 3 elements.

**Table 2:** Summary of the functional features of the 12 Fra-1-bound *TGFB2* enhancers

Enhancer	Requirement of Fra-1	Requirement of p300/CBP	Requirement of KAT activity	Enhancer category
+ 32	Yes	Yes	Yes	Dependent on Fra-1 and p300/CBP KAT activity
+ 115	Yes	Yes	Yes	Dependent on Fra-1 and p300/CBP KAT activity
+ 118	Yes	Yes	Yes	Dependent on Fra-1 and p300/CBP KAT activity
+ 136	Yes	Yes	No	Dependent on Fra-1 and p300/CBP but not on its KAT activity
+ 151	Yes	Yes	Yes	Dependent on Fra-1 and p300/CBP KAT activity
+ 240	Yes	Yes	Yes	Dependent on Fra-1 and p300/CBP KAT activity
+ 314	Yes	Yes	Yes	Dependent on Fra-1 and p300/CBP KAT activity
+ 315	Yes	Yes	Yes	Dependent on Fra-1 and p300/CBP KAT activity
+ 360	No	Yes	Yes	Fra-1-independent but dependent on p300/CBP KAT activity
+ 744	Yes	Yes	No	Dependent on Fra-1 and p300/CBP but not on its KAT activity
+ 980	Yes	No	No	Dependent on Fra-1 but p300/CBP-independent
+ 1426	No	Yes	No	Fra-1-independent, dependent on p300/CBP but not on its KAT activity

The positions of the enhancers with respect to the *TGFB2* TSS are indicated in kb. Dependences on Fra-1, p300/CBP and p300/CBP KAT activity for eRNA production are indicated for each enhancer

### Fra-1 differentially affects the recruitment of p300/CBP at the *TGFB2* enhancers

Next, we tested whether Fra-1 could operate via altering p300/CBP recruitment at the *TGFB2* enhancers. This was achieved in ChIP-qPCR assays conducted after RNAi-induced depletion of Fra-1. p300/CBP signals did not change except at 3 enhancers (+ 115 kb, + 136 kb and + 151 kb) where they significantly increased (Fig. 5C). An effect at only a fraction of the enhancers was in fact not surprising, as we formerly showed that Fra-1 knockdown modulates (positively and negatively) the binding of p300/CBP at only half of the 4129 Fra-1-bound candidate active enhancers found in the whole MDA-MB-231 cell genome [36]. The results on the *TGFB2* enhancers that were obtained during the course of this genome-wide study are presented in Additional file 6: Data S6C and showed coherent with our herein ChIP-qPCR data. Thus, despite its binding at all of the *TGFB2* enhancers, Fra-1 was found to be instrumental for limiting (directly or indirectly; see Discussion) p300/CBP recruitment at only one fourth of them.

### p300/CBP KAT activity is differentially required for Fra-1-regulated eRNA production at the *TGFB2* enhancers

Finally, we addressed whether the Fra-1-induced limitation of *TGFB2* expression involves p300/CBP enzymatic activity for the regulation of *TGFB2* enhancers.

In a first step, we tested whether cellular p300/CBP KAT activity is instrumental, not just for *TGFB2* expression in the presence of Fra-1 as shown in Fig. 5B, but also for enhanced transcription of *TGFB2* upon Fra-1 down-regulation. To this aim, MDA-MB-231 cells were transfected with either siFra-1 or siCTL for 72 h with A485 being added 56 h after the beginning of the transfection to inhibit p300/CBP enzymatic activity (i.e. at a time point where Fra-1 has already massively disappeared). Addition of the drug abrogated the increase in *TGFB2* mRNA steady-state level triggered by Fra-1 depletion (Fig. 5D, middle panel), indicating that Fra-1 limits by a still-to-be-identified mechanism the positive action of p300/CBP KAT activity on *TGFB2* expression in MDA-MB-231 cells.

As a next step, we asked whether p300/CBP KAT activity could be necessary for increased activity of *TGFB2* enhancers upon Fra-1 down-regulation. To this aim, we quantified eRNA production at all *TGFB2* enhancers after transfection of MDA-MB-231 cells with either siFra-1 or siCTL followed by inhibition of p300/CBP enzymatic activity by addition of A485 (Fig. 5D, right panel). The control experiments confirmed our former data on this production after simple knockdown of Fra-1 (Fig. 4A) and ruled out any major experimental bias linked to the presence of the A485 solvent (DMSO).

For 7 enhancers (+ 32 kb, + 115 kb, + 118 kb, + 151 kb, + 240 kb, + 314 kb and + 315 kb) where eRNA production was increased upon simple Fra-1 down-regulation (Fig. 4A) and decreased upon simple p300/CBP KAT activity inhibition (Fig. 5B, right panel), increased eRNA production triggered by Fra-1 knockdown was abrogated by A485 (Fig. 5D, right panel). This supported the idea that p300/CBP KAT activity is actually required for increased activity of these enhancers in the absence of Fra-1. It is also interesting to note that, despite binding to all of these 7 regulatory elements (Table 1), Fra-1 regulates (directly or indirectly) the abundance of p300/CBP at only 2 of them (+ 115 kb and + 151 kb) (Fig. 5C). As p300/CBP is present at the 7 enhancers (albeit in different amounts), this raised the idea that the regulations of p300/CBP recruitment and enzymatic activity at the different *TGFB2* enhancers may not necessarily be coupled for regulating *TGFB2* transcription (see Discussion).

The five other enhancers behaved differently. At position + 136 kb, eRNA production showed dependent on p300/CBP (Fig. 5A, right panel) but independent of p300/CBP KAT activity whether in the presence (Fig. 5B, right panel) or in the absence of Fra-1 (Fig. 5D, right panel) despite increased recruitment of p300/CBP after knockdown of Fra-1 (Fig. 5C). This further supported the possible uncoupling of p300/CBP recruitment and enzymatic activity regulation at the *TGFB2* enhancers. At position + 360 kb, dependence on p300/CBP KAT activity already seen in Fig. 5B (right panel) was also observed after Fra-1 down-regulation. This was not surprising, as enhancer activity at this site was previously shown independent of Fra-1 (Fig. 4A). Concerning enhancer + 744 kb, its decreased activity upon Fra-1 knockdown was not stronger when p300/CBP KAT activity was concomitantly abrogated (Fig. 5B and Fig. 5D). This confirmed the independence of this enhancer on p300/CBP enzymatic activity. Surprisingly, at enhancer + 980 kb, only a poor eRNA induction was detected upon knockdown of Fra-1. This result differed from our previous Fra-1 RNAi experiments (Fig. 4A) for reasons that are still unclear. Whatever the mechanism involved, p300/CBP KAT activity was however not found required for eRNA synthesis at this location, as already observed in the presence of Fra-1 (Fig. 5B). Finally, the result obtained at position + 1426 kb was puzzling. On the one hand, we confirmed that simple elimination of Fra-1 does not increase enhancer activity as formerly shown (Fig. 4A). However, on the other hand, we observed a dependence on p300/CBP KAT activity contrasting with the data shown in Figs. 5A and B (right panel), which was rather suggestive of a structural/organizational role for p300/CBP at this site in the presence of Fra-1. Further studies are required to understand how RNAi-mediated depletion of Fra-1 induces a shift from a non-catalytic requirement to a catalytic one for p300/CBP at this location.

Thus, our data show that increased *TGFB2* expression induced by Fra-1 down-regulation is dependent on the KAT activity of p300/CBP. However, the study of the *TGFB2* locus points to, not only differential ability for Fra-1 to permit the recruitment of p300/CBP at the different *TGFB2* enhancers, but also differential requirements upon its KAT activity for the functioning of these regulatory elements. It also suggests that the regulations of p300/CBP recruitment and enzymatic activity can be uncoupled, at least, at some of them. Taken together, these observations strengthen the notion of molecular and functional heterogeneity of the Fra-1-bound *TGFB2* enhancers.

## Discussion

**Fig. 6:**
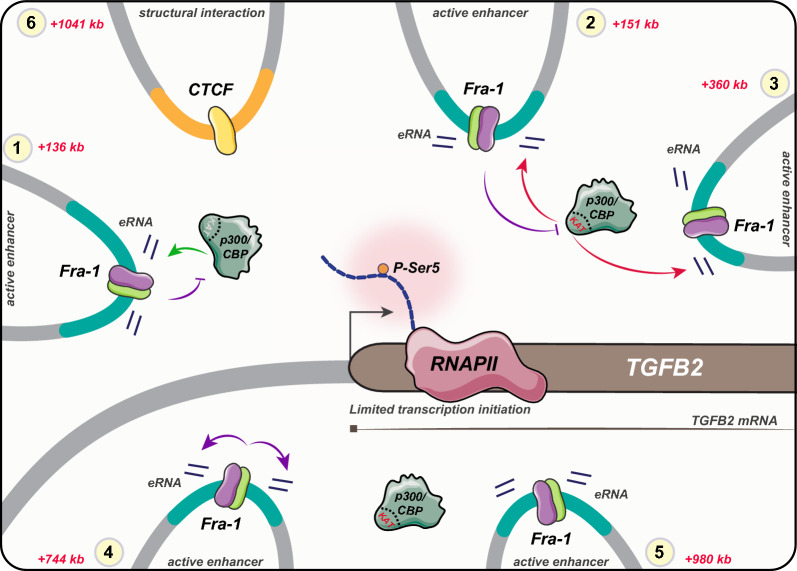
Schematic representation of the molecular heterogeneity and differential roles of Fra-1 and p300/CBP at the *TGFB2* PIRs. This model shows various types of PIRs: (1) Fra-1 modulates the recruitment of p300/CBP, whose presence but not the KAT activity is required for enhancer activity (e.g. enhancer at + 136 kb). (2) Fra-1 inhibits p300/CBP KAT activity which is important for enhancer activation (e.g. enhancer at + 151 kb), (3) enhancer activity depends on p300/CBP KAT activity but not on Fra-1 (e.g. enhancer at + 360 kb), (4) Fra-1 enhances eRNA production independently of p300/CBP KAT activity, (5) neither Fra-1 nor p300/CBP affects enhancer activity (e.g. enhancer at + 980 kb) and (6) CTCF-bound PIR contributing to the structural organization of this locus. The overall activity of Fra-1 at these enhancers inhibits Pol II-Ser5 phosphorylation, limiting transcription initiation at TGFB2 promoter. Fra-1 is indicated in purple and its dimerization partner in green. Violet arrows indicate the positive/negative effects of Fra-1 whereas the green and red arrows indicate KAT-independent- and KAT-dependent effects of P300/CBP in eRNA production, respectively

How Fra-1 molecularly regulates transcription has little been addressed thus far. This contrasts with its many described physiological and pathological functions, including in cancer where Fra-1 is the most frequently implicated member of the Fos family [8–12, 69]. In particular, it is not known how it represses its target genes via binding to distant regulatory elements, the latter being by far more numerous than promoter-proximal ones [24, 36]. As a first step towards clarifying this issue, we have taken advantage of the strong down-regulation of the *TGFB2* gene by Fra-1 in the MDA-MB-231 TNBC model, as well as of the remote locations of the candidate enhancers of this gene. Taken together, our data point to a complex situation with multiple effects of Fra-1 on the *TGFB2* regulatory elements it binds to with differential implications of the lysine acetyl transferase p300/CBP (Fig. 6).

Our work indicates that *TGFB2* repression by Fra-1 is achieved, at least in part, transcriptionally via decreasing the formation of the transcription-initiating form of Pol II without affecting the recruitment of the polymerase at the gene promoter. As Fra-1 stimulates the transcription of the *HMGA1* gene via facilitating Pol II recruitment at its promoter [48] and that of the *PLAU* gene via facilitating the formation of the transcription-elongating form of Pol II (phosphorylated on CTD Ser2) [47] in MDA-MB-231 cells, our observation points to the important notion that the molecular effects of Fra-1 on Pol II can be multiple with possibly opposite transcriptional outcomes, including in the same cell context. However, to which extent these effects are direct or indirect remains to be established.

Here, we have shown that the *TGFB2* promoter interacts with 10 PIRs that contain at least one Fra-1-bound enhancer. Moreover, we also report that the formation of the chromatin loops between the *TGFB2* promoter and its enhancers is not modulated by Fra-1. It is therefore most likely that Fra-1 exploits pre-exiting chromatin loops to prevent the formation of the transcription-initiating form of Pol II and exert its repressive effects. Importantly, our data indicate that the decrease in phosphorylation of Pol II CTD Ser5 is not linked to a decrease in CDK7 recruitment that would be mediated by Fra-1. Coherently with this observation, recent proteomic studies conducted in another TNBC cell line (BT549) [41] and in human Th17 cells [70] did not reveal any association of Fra-1 and CDK7 within supramolecular complexes. One possibility to explain how Fra-1 regulates RPB1 CTD Ser5 phosphorylation might consequently be modulation of CDK7 activity via a yet-to-be-identified factor. However, it cannot be ruled out that Fra-1 may also enhance the recruitment of (or upregulate) a RPB1 CTD PSer5-specific phosphatase or decrease the recruitment of (or inhibit) a kinase capable of phosphorylating RPB1 CTD Ser5. Future work is required to solve this issue.

Besides the transcriptional contribution of Fra-1 to the down-regulation of *TGFB2* expression, our run-on- and pre-mRNA level assays suggest that there could also exist a post-transcriptional one. This possibility is all the more to be taken into consideration that Fra-1 has been shown to regulate the expression of various miRNAs that may affect the stability of other RNAs in other cell contexts [71, 72].

Several of our data suggest that *TGFB2* gene regulation depends on complex chromatin 3D organization and/or dynamics within the *TGFB2* TAD. Thus, the *TGFB2* promoter interacts with 15 PIRs scattered from one to the other extremity of this TAD with most of them being located far away from the *TGFB2* gene's TSS (i.e. from 116 kb up to 1450 kb for 13 of them). Moreover, amongst the PIRs, (i) 8 harbor Fra-1-bound active enhancers, (ii) 4 harbor CTCF-bound candidate chromatin 3D structure-organizing domains, (iii) 2 harbor at least one CTCF-bound candidate chromatin 3D structure-organizing domain plus a Fra-1-bound active enhancer, and (iv) one bears a possible Epromoter. Finally, the PIRs are not clustered according to their types (i.e. enhancer activity, chromatin organization or putative Epromoter) but are rather tangled, especially in the most distal moiety of the *TGFB2* TAD. In this context, it is interesting to note that several genes are located between the *TGFB2* PIRs or overlap certain of them but are not regulated by Fra-1, which suggests the existence of regulation mechanisms capable of discriminating between them and *TGFB2*. While these observations point to a complex regulatory scheme in this particular region of the genome, they unfortunately do not allow to propose a precise overall functioning for it, as the Hi-C-, NG Capture-C- and ChIP-seq techniques used in our study give averaged frequencies in chromatin interactions or markings occurring in a whole cell population but do not provide any insight into their stability, dynamics, simultaneity or succession over time in individual cells. Further fine molecular studies will therefore be necessary to understand why Fra-1 regulates the *TGFB2* gene but not the other genes residing within the *TGFB2* TAD. Whatever these mechanisms might be, our NG Capture-C experiments indicate that they do not involve gross Fra-1-dependent chromatin rearrangements at the *TGFB2* locus.

Our data indicate that Fra-1 represses *TGFB2* by binding exclusively to active enhancers that all bear at least one AP-1-binding site. We consider this observation important for two reasons. On one side, it excludes the possibility that Fra-1 exerts its repressive effect on *TGFB2* expression via binding to silencer elements [73] distinct from Fra-1-non-recruiting enhancers that would stimulate *TGFB2* transcription. On the other side, it strongly supports the idea that Fra-1 attenuates the activity of multiple enhancers collaborating to *TGFB2* expression through direct binding to them. As already mentioned, this negative effect of Fra-1 cannot be explained by induction of major changes in the chromatin 3D organization of the *TGFB2* locus and our ATAC-seq data indicate that they do not involve marked changes in local chromatin accessibility either.

Further work will therefore have to establish whether Fra-1 binds to discrete autonomous silencer elements embedded within Fra-1-bound *TGFB2* enhancers or limits the activity of the latter via other mechanisms. The first possibility is challenging to address, as silencers still constitute particularly ill-understood genomic entities [73], and the second is all the more to be considered that there is accumulating evidence that enhancers may, in fact, be two-faceted genetic elements endowed with interwoven transcription *trans*-activating- and -*trans*-repressing activities, the contributions of which may vary according to the cell and/or signaling context [73].

Interestingly, several of our observations suggest that the role of Fra-1 at the regulatory elements it binds to is likely pleiotropic. Firstly, the various Fra-1-bound active enhancers show strong molecular heterogeneity, as indicated by marked differences in Fra-1-, p300/CBP-, H3K4me1-, H3K4me3-, H3K27ac- and Pol II ChIP-seq- and ATAC-seq signal intensities. Secondly, assessing enhancer activity on the basis of their ability to produce eRNAs showed that, among the 12 Fra-1-bound candidate active enhancers, 9 of them have their activity repressed by Fra-1, 2 were unaffected by Fra-1 and 1 was even stimulated by Fra-1. This, not only provides additional support to the idea of functional heterogeneity for the Fra-1-bound *TGFB2* enhancers, but also suggests that the final *TGFB2* transcriptional output may result from a balance between repressive, neutral and positive actions of Fra-1 at these 3 categories of enhancers, respectively. Thirdly, our data also strongly point to heterogeneity amongst the Fra-1-bound *TGFB2* enhancers with respect to their dependence upon p300/CBP presence and KAT activity for eRNA production, as well as to different implications of Fra-1 at these regulatory elements. Thus, we show that the activity of one of the 12 Fra-1-bound enhancers is independent of the presence of p300/CBP even though p300/CBP binds to it. Moreover, among the 11 p300/CBP-dependent enhancers, only 8 of them require the KAT activity, suggesting that p300/CBP may only play a structural/organizational role at the remaining other 3 elements. The latter possibility was not unexpected, as enzymatic activity-independent scaffolding properties have regularly been reported for p300/CBP [65–68]. Finally, Fra-1 was found to be instrumental for limiting p300/CBP recruitment at only one fourth of the enhancers it binds to. Thus, taken together, our data support the notion of molecular and functional heterogeneity for the Fra-1-bound *TGFB2* enhancers in a general context where the biochemical, epigenetic, operational and functional definition of enhancers is a matter of intense debate [1–3].

The role of p300/CBP at the *TGFB2* enhancers in MDA-MB-231 cells deserves additional comments. It has been reported that the RNAs transcribed proximally to p300/CBP-binding sites in chromatin (at the forefront of which are eRNAs) stimulate the KAT activity of p300/CBP, leading to enhanced acetylation of histones and, thereby, increased transcription of target genes [74]. Our observation that the presence of p300/CBP is a prerequisite for eRNA production at 11 of the 12 Fra-1-bound *TGFB2* enhancers with KAT activity being necessary for only 8 of them suggests that this model is not universal and also raises the question of the molecular role of p300/CBP in the activity of these enhancers. In particular, for the 8 enhancers where KAT activity is required for eRNA production, it will be interesting to identify which of the many possible p300/CBP non-histone substrates [66–68, 75] is/are instrumental for *TGFB2* enhancer activity and gene transcription. Additionally, it will also be interesting to elucidate how Fra-1 limits eRNA production at 2 of these enhancers where it reduces the recruitment of p300/CBP, as well as at those where it decreases KAT activity. In the latter case, indirect mechanisms will have to be considered as, even though Fra-1 and p300 have been shown co-immunoprecipitable in cell transfection experiments in one study [46], the proteomic studies already mentioned above did not reveal any association between endogenous p300/CBP and Fra-1 in TNBC- and human Th17 cells [41, 70].

Another question raised by our work is whether the Fra-1-bound *TGFB2* enhancers function collectively through the formation of transcriptional hubs whose importance is increasingly documented in the literature [4, 76], including in the case of AP-1-regulated genes [8, 39]. As mentioned earlier, our NG Capture-C experiments only give a statistical view of the interaction frequencies between the *TGFB2* promoter and the various chromatin regions it can interact with in a whole cell population. However, it gives no indication on the number of PIRs that are in contact with other PIRs and/or with the *TGFB2* promoter in each cell at a given time, as well as on whether these interactions are stochastic or sequentially ordered. Moreover, if our eRNA assays suggest that all Fra-1-bound *TGFB2* enhancers actually possess some transactivation potential, they do not indicate whether certain of them are more potent than others at transactivating the *TGFB2* gene (in the presence or in the absence of Fra-1). Future work will therefore have to characterize the individual contributions of each one of these regulatory elements to *TGFB2* transcriptional regulation. In particular, it will have to decipher their collective *versus* redundant functioning, as well as to establish whether they come into play concurrently or dependently on structural and/or sequential constrains.

A next important issue concerns how the diverse mechanisms used by Fra-1 at the various Fra-1-bound *TGFB2* enhancers could converge to reduce transcription initiation at the *TGFB2* promoter. There is accumulating evidence that liquid–liquid phase separation-induced biomolecular condensates incorporating gene promoters and enhancers are essential for transcription to occur and that gene regulation may involve controlling their assembly/dissolution [77, 78]. As condensate formation is thought to be largely dependent on the binding of multiple TFs to enhancers, a first straightforward possibility might be that the binding of Fra-1 to AP-1 binding-sites at *TGFB2* enhancers would perturb the crowding of the other TFs at these enhancers and, thereby, would lead to rearrangements within these condensates. As RNAs [77, 78], and particularly eRNAs [79], produced within these structures are thought essential for the formation/dissolution of transcription condensates, another non-exclusive possibility could be that repression of eRNA transcription by Fra-1 at most Fra-1-bound *TGFB2* enhancers would also affect the organization of *TGFB2* transcriptional condensates. Interestingly, it has recently been proposed that, rather than tight physical contacts between promoters and their cognate enhancers, sufficient proximity would be sufficient for molecular communication to occur between these gene regulatory elements [80]. In this model, which better accounts for a number of microscopy-based observations made on live cells, communication would be ensured by distance-dependent diffusion of transcription factors and co-factors from enhancers towards promoters. Should this apply to *TGFB2* in MDA-MB-231 cells, it would be important to identify the molecules trafficking from its enhancers to its promoter within transcriptional condensates and to elucidate whether and how Fra-1 alters their movements.

As Fra-1 does not bind to DNA as an homodimer [6, 8], another question concerns the nature and the role of Fra-1 heterodimerization partner(s) in the repression of *TGFB2*. This issue is, in fact, complex and we cannot exclude that multiple partners come into play at the various *TFGB2* candidate enhancers for various reasons: (i) even though proteomic studies have shown that Fra-1 can heterodimerize with the 3 members of the Jun family in TNBC cells [41], it must be taken into account that Fra-1 can also heterodimerize with non-Jun proteins [6, 8], (ii) Fra-1-containing AP-1 dimers most likely turn over rapidly at AP-1-binding sites in TNBCs [36], (iii) AP-1 dimers constantly form and dissociates in vivo with interaction times of less than a few minutes [81] and (iv) it is possible that different dimer(s) may be required at different candidate enhancers. Whatever the partner(s) involved, the fact that we observed enhanced transcription of *TGFB2* upon simple RNAi-mediated down-regulation of Fra-1 indicates that Fra-1 plays a crucial role in the concerned AP-1 dimers. This observation is consistent with the fact that Fra-1 was described to exert a prominent part, not only in the transcriptional activity of the AP-1 dimers where it is involved, but also in AP-1-binding site selection [82].

## Conclusion

We report here that Fra-1 can down-regulate one of its target genes via binding to multiple enhancers showing different molecular and functional features. Interestingly, some of its actions are even antagonistic since the final *TGFB2* expression outcome results from an equilibrium between enhancers negatively or positively regulated by Fra-1. This highlights the important notion that the molecular actions of Fra-1 bound to multiple enhancers for down-regulation of the same gene can be heterogeneous. As most gene promoters interact with several enhancers [1–5], we feel important to consider that what we have observed for Fra-1 on *TGFB2* may also apply to other Fra-1-down-regulated genes, as well as to other TFs capable of binding several enhancers of the same gene. Among the latter, the other Fos and Jun components of the ubiquitous AP-1 transcription complex must be considered in priority, as recent genome-wide transcriptomic and genomic investigations have shown that several Fos and Jun family proteins can nearly equivalently up- or down-regulate gene expression in the same cellular context [8]. Future gene-specific studies should elucidate how diverse mechanisms at play at the different enhancers of the same gene may collaborate, whether it is AP-1- or non-AP-1 transcription factors/complexes that are involved in gene expression down-regulation.

## Materials and methods

### Cell culture

MDA-MB-231 cells were obtained from the ATCC. Cells were cultured at 37 °C in DMEM supplemented with 10% fetal calf serum and penicillin/streptomycin (100 µg/ml each) in a humidified, 5% CO_2_-containing atmosphere. They were routinely tested for the absence of mycoplasma contamination.

### RNAi

siRNAs were transfected at a final concentration of 5 nM for 72 h using INTERFERin (Polyplus) according to the supplier’s specifications. For RNAi-mediated depletion of Fra-1, a pool of 3 siRNAs (siFra-1) was used as described in Tolza et al. [48] to provide high specificity and minimize off-target effects. The control siRNA (siCTL) and the siRNAs against p300 (sip300) and CPB (siCBP) were obtained from ThermoFisher. siRNA sequences or references are given in Additional file 7: Table S1A.

### Antibodies

The anti-Fra-1- (sc-376148X and sc-28310X),—Pol II- (sc-55492X) and -GAPDH (sc-25778) antibodies were from Santa Cruz Biotechnology, as well as control IgG (sc-2025). The anti-p300- (D2X6N, #54062) -CBP (D6C5, #7389) and -CDK7 (#2916) antibodies were from Cell Signaling Technology. The anti-Pol II-PSer5 antibody (clone H14, # 920304) was from Biolegend. The mouse anti-IgM antibody (04–6800) was from Invitrogen. The anti-H3K36me3 antibody (61101) was from Active Motif and the anti-H3 antibody (05–928) from Millipore. The anti-H3K27ac (Ab4729) and -p300/CBP antibodies (Ab14984) were from Abcam. The anti-rabbit (sc-2313) and anti-mouse (sc-2954) horseradish peroxidase (HRP)-conjugated secondary antibodies were from Santa Cruz Biotechnology.

### Immunoblotting

Immunoblotting experiments were performed as described previously [47] and proteins were detected using the Luminata Forte Western HRP kit from Millipore.

### p300/CBP HAT activity inhibition

Cellular p300/CBP HAT activity was inhibited by adjusting the cell culture medium to 3 nM of A485 (Tocris, #6387) using a stock solution prepared in DMSO at a concentration of 5 mM. In control samples, an equivalent volume of DMSO was added to the culture medium.

### Run-on assays, RNA extraction, reverse transcription and quantitative PCR (qPCR)

Transcriptional run-ons, RNA extraction, reverse transcription and qPCR were performed as described previously [48]. For RT-qPCR, 1 and 2.5 µg of purified total RNAs were used for the analysis of mRNA and eRNA abundance, respectively. The primers used for qPCR assay of mRNAs, nascent RNAs and pre-mRNAs are given in Additional file 7: Table S1B. The primers used for eRNA amplification are given in Additional file 7: Table S1C.

### ChIP-qPCR

ChIP-qPCR were performed as described previously [48]. The sequences of the primers used to amplify the different amplicons of the *TGFB2* gene are given in Additional file 7: Table S1D.

### ATAC-seq

ATAC-seq was performed according to Buenrostro et al. [83]. Briefly, 3 biological replicates were used for each condition using 100,000 MDA-MB-231 cells transfected with either siCTL or siFra-1. After 72 h, cells were harvested by centrifugation and washed once with PBS. The pellets were resuspended in 100 µl of cold Cell Lysis Buffer (Tris–HCl pH 7.5 10 mM, NaCl 10 mM, MgCl_2_ 3 mM and Igepal CA-630 0.1%). Lysed cells were centrifuged at 500 g at 4 °C for 10 min and the pellets were then resuspended in 100 µl of the Transposase reaction mix (50 µl of 2X TD Buffer from Illumina, cat. n° 15027866) to which 5 µl Tn5 transposase (Illumina, cat. n° 15027865) and 45 µl of Nuclease-free water were added before an incubation of 30 min at 37 °C. DNA was then purified using MinElute Qiagen columns (Qiagen cat. n° 28004) according to the manufacturer’s instructions. At this stage, libraries were indexed using the Phusion^®^ High-Fidelity DNA Polymerase (NEB cat. n° M0530). Sequencing was performed using NovaSeq 6000 (Illumina) in paired-end (50 nt). Reads were aligned to the hg19 *Homo sapiens* genome using Bowtie2 and, then, processed using the R package PASHA [84]. The three biological replicates were then merged using UCSC bigWigMerge tool.

### FAIRE-qPCR

Formaldehyde-assisted isolation of regulatory elements (FAIRE) was performed as described by Rodriguez-Gil et al. [63]. Briefly, 2 × 10^6^ MDA-MB-231 cells were transfected with either siCTL or siFra-1. 72 h later, cells were fixed at 25 °C for 7 min using 1% paraformaldehyde (Euromedex). Fixation was stopped by addition of glycine 2.5 mM. Cells were then resuspended in 1 ml of Cell Lysis Buffer (PIPES pH7.5 5 mM, KCl 85 mM, NP40 0.5% and Na butyrate 10 mM containing 10 μg/ml of aprotinin, leupeptine, pepstatin and 250 μg/ml of AEBSF) and let on ice for 10 min. Nuclei were recovered by mild centrifugation at 4 °C and lysed in 250 µl of Nuclei Lysis Buffer (Tris–HCl pH7.5 50 mM, SDS 0.25%, EDTA 10 mM, Na butyrate 10 mM and protease inhibitors as in the cell lysis buffer) at 4 °C for 2 h. They were then sonicated at 4 °C using 10–15 cycles (up to clarification) of the Bioruptor Pico device from Diagenode. After centrifugation at 13,000 g at 4 °C for 15 min, samples were split into two 100 µl aliquots. One of the aliquots, which constituted the total DNA reference, was decrosslinked at 65 °C for 6 h after successive RNAse A and Proteinase K treatments and DNA was purified by phenol/chloroform extraction following the procedures described in ref. [63]. The other aliquot was only RNAse A-treated before free DNA (i.e. DNA carrying gene regulatory regions showing no strong association with protein complexes such as nucleosomes) was purified by phenol/chloroform extraction. DNA quantification by qPCR, as well as determination of free *versus* total DNA amounts, were also performed as described in ref. [63]. Primers used to amplify the different amplicons on the *TGFB2* locus are given in Additional file 7: Table S1D.

### NG capture-C and PeakC analysis

NG Capture-C experiments and peak calling analyses are described in detail in Bejjani et al. [36]. Peak calling was carried out using the PeakC R package [53] using the following parameters: alphaFDR = 0.1, wSize = 5 and qWr = 1. Adjacent restriction fragments called by PeakC were then merged into a single region using Bedtools, thus defining PIR sizes. TAD boundaries were defined by Rao et al. [55] using an Arrowhead transformation.

### Statistical analyses

Data were reported as the means ± SEM and analyzed by unpaired two-tailed Student’s t-test or ANOVA depending on the number of samples being compared (2 or more than 2, respectively) with the GraphPad Prism5 software. P-values were considered as significant when *, ≤ 0.05 and highly significant when **, ≤ 0.01; ***, ≤ 0.005; ****, ≤ 0.001).

## Supplementary Information

**Additional file 1: Data S1.** Transcriptional repression of *TGFB2* by Fra-1. The experiments presented in (A), (B) and (C) are the same as those presented in Figure 1B, -C and -D, except that other amplicons were used. The sequences of the oligonucleotides used in RT-qPCR assays are given in Additional file 7: Table S1B. (D) *RPS26 gene and ChIP-qPC*R. The upper panel shows the amplicon positions used in ChIP-qPCR experiments, indicated in kb from the TSS. The middle panel shows ChIP-qPCR analysis of total Pol II on the RPS26 gene (n=4) and the lower panel shows ChIP-qPCR analysis of Pol II-PSer5 on the RPS26 gene (n=4). All values were normalized to that of amplicon − 0.8 kb under control condition arbitrarily set to 1. The arrows indicate the RPS26 TSS. All experiments were carried out using MDA-MB-231 cells transfected with either siFra-1 (green boxes) or siCTL (violet boxes) for 72 hours. (E) *Expression levels of the genes located in the TGFB2 TAD upon Fra-1 down-regulation*. The transcriptome regulated by Fra-1 was formerly identified using Affymetrix GeneChip Human Gene 2.0 ST arrays [36]. Gene expression ratios in siFra-1 *versus* siCTL conditions showed that the mRNA steady-state levels *of RRP15, LYPLAL1* and *LINC02869* were not affected upon Fra-1 down-regulation. Expression of *LYPLAL1*-*DT*, *LYPLAL1*-*AS1*, *ZC3H11B* and *LINC01710* could not be analyzed (NA), as the corresponding probes were absent from the Affymetrix arrays used.

**Additional file 2: Data S2**. Positions of AP-1 motifs under the Fra-1 ChIP-seq peaks at the Fra-1-bound *TGFB2* enhancers. The positions of the Fra-1-bound enhancers are given with respect to the TGFB2 TSS. AP-1 motif positions are given both with respect to the TGFFB2 TSS and in the reference genome Hg19. The sequences of the AP-1- and AP-1-related motifs are indicated in black and blue, respectively.

**Additional file 3: Data S3.** Molecular features of the *TGFB2* PIRs. ChIP-seq data for H3K4me3, H3K27ac, H3K4me1, Pol II, CTCF, p300/CBP, Fra-1, as well as ATAC-seq data, were used to, not only classify the 15 *TGFB2* PIRs defined by NG Capture-C in MDA-MB-231 cells, but also show their molecular heterogeneity. The first category contains the PIRs harboring only a Fra-1-bound candidate active enhancer (PIRs +32, +136, + 151, +240, +360 and +980). These are all characterized by overlapping Fra-1- and ATAC-seq signals and absence of CTCF and H3K4me3 signals. However, they show some heterogeneity in H3K4me1, H3K27ac, Pol II, and p300/CBP signal intensities. The second category contains PIRs marked by CTCF but not by Fra-1 (PIR -46, +1041, +1222 and +1316). At variance with Fra-1-bound active enhancers, CTCF-bound elements are not associated with ATAC-seq signals. The third category corresponds to a PIR (PIR +836) that is not bound by Fra-1 but bears an active gene promoter (open chromatin configuration in ATAC-seq experiments and strongly marked by H3K4me1, H3K4me3 and H3K27ac) with possible enhancer function for *TGFB2* (Epromoter). The fourth category contains PIRs (PIR +116 and +314) that carry two Fra-1-bound active enhancers specified by overlapping Fra-1- and ATAC-seq signals. Like in the first category of PIRs, the candidate enhancers are not marked by CTCF and H3K4me3 and show some heterogeneity in H3K4me1, H3K27ac, Pol II, and p300/CBP signals. Finally, the fifth category (PIRs +729 and +1449) contains PIRs bearing one Fra-1-bound active enhancer, as well as at least one CTCF-binding elements.

**Additional file 4: Data S4.** Bidirectional transcription at the *TGFB2* locus in MDA-MB-231 cells. Nascent RNA production was assessed by others in MDA-MB-231 cells using Gro-seq [62]. The publicly available Gro-seq data (upper panel) were aligned along with the *TGFB2* locus NG Capture-C data (lower panel), as well as Fra-1 ChIP-seq data obtained in Bejjani et al. [36]. Purple signals indicate sense (+) transcription with respect to *TGFB2* gene transcription and orange ones antisense (-) transcription.

**Additional file 5: Data S5.** assay of eRNAs at the +32 Fra-1-bound PIR. (A) *Molecular features of intron 2 from position 218,536,777 to 218,577,587*. The Fra-1-binding site located at +32 kb resides in a region of open chromatin (ATAC-seq signal) also recruiting p300/CBP and marked by H3K4me1 and H3K27ac but not by CTCF. In contrast, there is no sign of open chromatin at positions +21.6 kb and +55.8 kb, as well as of marks specifying active enhancers. The amplicons used to assay eRNA versus TGFB2 pre-mRNA at position +32 kb are indicated by black boxes. (B) *RT-qPCR assay of eRNA versus TGFB2 pre-mRNA at position +32 kb in MDA-MB-231 cells.* RNA levels at positions +21.6-, 32- and 55.8 kb from the *TGFB2* TSS were quantified by RT-qPCR. RNA amplified at position +21.6- and +55.8 kb correspond to pre-mRNA, whereas RNA amplified at position +32 kb corresponds to pre-mRNA+eRNA. The data are the results of 6 independent experiments. Relative RNA abundances at the 3 positions were calculated by the ∆∆CT method, amplicon +32 being taken as the reference. Primer sequences for qPCR amplification are given in Additional file 7: Table S1B.

**Additional file 6: Data S6.** ATAC-seq signal ratios obtained in the absence and presence of Fra-1 at the Fra-1-bound *TGFB2* enhancers. Three independent ATAC-seq experiments were carried out. (A) The ratios of ATAC-seq signal intensities in siFra-1 *versus* siCTL conditions are given for the 12 Fra-1-bound enhancers, as well as for the *TGFB2* promoter. The start and end positions of the ATAC-seq peaks are given as well as the peak positions relative to the *TGFB2* TSS. (B) The ratios of ATAC-seq signal intensities in siFra-1 *versus* siCTL conditions are given for two peaks (peak A on chromosome 16 and peak B on chromosome 15) for which the ATAC-seq signals were found increased or decreased (FC≥±1.5), respectively. The start and end position of peaks are given in the table. (C) The ratios of p300/CBP signal intensities in siFra-1 *versus *siCTL conditions at the Fra-1-bound *TGFB2* enhancers showing a FC ≥ ±1.5 are indicated. ChIP-seq data obtained in MDA-MB-231 cells were previously presented in Bejjani et al. [36]. The peak positions relative to the *TGFB2* TSS, as well as the start and end positions of the p300/CBP peaks are given.

**Additional file 7.** Additional Tables.

## Data Availability

The ChIP-seq data for H3K4me3, H3K4me1, H3K27Ac, p300/CBP, Pol II and CTCF in MDA-MB-231 cells are available on the GEO database, Accession number GSE146822. https://www.ncbi.nlm.nih.gov/geo/query/acc.cgi?acc=GSE146822. Fra-1 ChIP-seq data in MDA-MB-231 cells are available on GEO database, Accession number GSE132098. https://www.ncbi.nlm.nih.gov/geo/query/acc.cgi?acc=GSE132098. NG-capture C data in MDA-MB-231 cells are available on the GEO database, Accession number GSE146824. https://www.ncbi.nlm.nih.gov/geo/query/acc.cgi?acc=GSE146824. H3K36me3 ChIP-seq data in MDA-MB-231 cells and ATAC-seq data in MDA-MB-231 cells transfected with either the control siRNA or the siRNA directed against Fra-1 are available on the GEO database, Accession number GSE208443.

## References

[1] Benton ML, Talipineni SC, Kostka D, Capra JA (2019). Genome-wide enhancer annotations differ significantly in genomic distribution, evolution, and function. BMC Genom.

[2] Andersson R, Sandelin A (2020). Determinants of enhancer and promoter activities of regulatory elements. Nat Rev Genet.

[3] Gasperini M, Tome JM, Shendure J (2020). Towards a comprehensive catalogue of validated and target-linked human enhancers. Nat Rev Genet.

[4] Di Giammartino DC, Polyzos A, Apostolou E (2020). Transcription factors: building hubs in the 3D space. Cell Cycle Georget Tex.

[5] Hamamoto K, Fukaya T (2022). Molecular architecture of enhancer-promoter interaction. Curr Opin Cell Biol.

[6] Chinenov Y, Kerppola TK (2001). Close encounters of many kinds: Fos-Jun interactions that mediate transcription regulatory specificity. Oncogene.

[7] Lopez-Bergami P, Lau E, Ronai Z (2010). Emerging roles of ATF2 and the dynamic AP1 network in cancer. Nat Rev Cancer.

[8] Bejjani F, Evanno E, Zibara K, Piechaczyk M, Jariel-Encontre I (2019). The AP-1 transcriptional complex: local switch or remote command?. Biochim Biophys Acta Rev Cancer.

[9] Young MR, Colburn NH (2006). Fra-1 a target for cancer prevention or intervention. Gene.

[10] Verde P, Casalino L, Talotta F, Yaniv M, Weitzman JB (2007). Deciphering AP-1 function in tumorigenesis: fra-ternizing on target promoters. Cell Cycle Georget Tex.

[11] Dhillon AS, Tulchinsky E (2015). FRA-1 as a driver of tumour heterogeneity: a nexus between oncogenes and embryonic signalling pathways in cancer. Oncogene.

[12] Talotta F, Casalino L, Verde P (2020). The nuclear oncoprotein Fra-1: a transcription factor knocking on therapeutic applications’ door. Oncogene.

[13] Jiang X, Xie H, Dou Y, Yuan J, Zeng D, Xiao S (2020). Expression and function of FRA1 protein in tumors. Mol Biol Rep.

[14] Young MR, Nair R, Bucheimer N, Tulsian P, Brown N, Chapp C (2002). Transactivation of Fra-1 and consequent activation of AP-1 occur extracellular signal-regulated kinase dependently. Mol Cell Biol.

[15] Terasawa K, Okazaki K, Nishida E (2003). Regulation of c-Fos and Fra-1 by the MEK5-ERK5 pathway. Genes Cells Devoted Mol Cell Mech.

[16] Belguise K, Milord S, Galtier F, Moquet-Torcy G, Piechaczyk M, Chalbos D (2012). The PKCθ pathway participates in the aberrant accumulation of Fra-1 protein in invasive ER-negative breast cancer cells. Oncogene.

[17] Belguise K, Cherradi S, Sarr A, Boissière F, Boulle N, Simony-Lafontaine J (2017). PKCθ-induced phosphorylations control the ability of Fra-1 to stimulate gene expression and cancer cell migration. Cancer Lett.

[18] Casalino L, De Cesare D, Verde P (2003). Accumulation of Fra-1 in ras-transformed cells depends on both transcriptional autoregulation and MEK-dependent posttranslational stabilization. Mol Cell Biol.

[19] Vial E, Marshall CJ (2003). Elevated ERK-MAP kinase activity protects the FOS family member FRA-1 against proteasomal degradation in colon carcinoma cells. J Cell Sci.

[20] Basbous J, Chalbos D, Hipskind R, Jariel-Encontre I, Piechaczyk M (2007). Ubiquitin-independent proteasomal degradation of Fra-1 is antagonized by Erk1/2 pathway-mediated phosphorylation of a unique C-terminal destabilizer. Mol Cell Biol.

[21] Talotta F, Mega T, Bossis G, Casalino L, Basbous J, Jariel-Encontre I (2010). Heterodimerization with Fra-1 cooperates with the ERK pathway to stabilize c-Jun in response to the RAS oncoprotein. Oncogene.

[22] Sayan AE, Stanford R, Vickery R, Grigorenko E, Diesch J, Kulbicki K (2012). Fra-1 controls motility of bladder cancer cells via transcriptional upregulation of the receptor tyrosine kinase AXL. Oncogene.

[23] Desmet CJ, Gallenne T, Prieur A, Reyal F, Visser NL, Wittner BS (2013). Identification of a pharmacologically tractable Fra-1/ADORA2B axis promoting breast cancer metastasis. Proc Natl Acad Sci USA.

[24] Zhao C, Qiao Y, Jonsson P, Wang J, Xu L, Rouhi P (2014). Genome-wide profiling of AP-1-regulated transcription provides insights into the invasiveness of triple-negative breast cancer. Cancer Res.

[25] Diesch J, Sanij E, Gilan O, Love C, Tran H, Fleming NI (2014). Widespread FRA1-dependent control of mesenchymal transdifferentiation programs in colorectal cancer cells. PLoS ONE.

[26] Iskit S, Schlicker A, Wessels L, Peeper DS (2015). Fra-1 is a key driver of colon cancer metastasis and a Fra-1 classifier predicts disease-free survival. Oncotarget.

[27] Suzuki T, Okuno H, Yoshida T, Endo T, Nishina H, Iba H (1991). Difference in transcriptional regulatory function between c-Fos and Fra-2. Nucleic Acids Res.

[28] Yoshioka K, Deng T, Cavigelli M, Karin M (1995). Antitumor promotion by phenolic antioxidants: inhibition of AP-1 activity through induction of Fra expression. Proc Natl Acad Sci USA.

[29] Belguise K, Kersual N, Galtier F, Chalbos D (2005). FRA-1 expression level regulates proliferation and invasiveness of breast cancer cells. Oncogene.

[30] Rajasekaran S, Reddy NM, Zhang W, Reddy SP (2013). Expression profiling of genes regulated by Fra-1/AP-1 transcription factor during bleomycin-induced pulmonary fibrosis. BMC Genom.

[31] Hasenfuss SC, Bakiri L, Thomsen MK, Williams EG, Auwerx J, Wagner EF (2014). Regulation of steatohepatitis and PPARγ signaling by distinct AP-1 dimers. Cell Metab.

[32] Bakiri L, Macho-Maschler S, Custic I, Niemiec J, Guío-Carrión A, Hasenfuss SC (2015). Fra-1/AP-1 induces EMT in mammary epithelial cells by modulating Zeb1/2 and TGFβ expression. Cell Death Differ.

[33] Gallenne T, Ross KN, Visser NL, Salony N, Desmet CJ, Wittner BS (2017). Systematic functional perturbations uncover a prognostic genetic network driving human breast cancer. Oncotarget.

[34] Maurus K, Hufnagel A, Geiger F, Graf S, Berking C, Heinemann A (2017). The AP-1 transcription factor FOSL1 causes melanocyte reprogramming and transformation. Oncogene.

[35] Hannemann N, Cao S, Eriksson D, Schnelzer A, Jordan J, Eberhardt M (2019). Transcription factor Fra-1 targets arginase-1 to enhance macrophage-mediated inflammation in arthritis. J Clin Invest.

[36] Bejjani F, Tolza C, Boulanger M, Downes D, Romero R, Maqbool MA (2021). Fra-1 regulates its target genes via binding to remote enhancers without exerting major control on chromatin architecture in triple negative breast cancers. Nucleic Acids Res.

[37] Marques C, Unterkircher T, Kroon P, Oldrini B, Izzo A, Dramaretska Y (2021). NF1 regulates mesenchymal glioblastoma plasticity and aggressiveness through the AP-1 transcription factor FOSL1. eLife.

[38] Chronis C, Fiziev P, Papp B, Butz S, Bonora G, Sabri S (2017). Cooperative binding of transcription factors orchestrates reprogramming. Cell.

[39] Phanstiel DH, Van Bortle K, Spacek D, Hess GT, Shamim MS, Machol I (2017). Static and dynamic DNA loops form AP-1-bound activation Hubs during macrophage development. Mol Cell.

[40] Fonseca GJ, Tao J, Westin EM, Duttke SH, Spann NJ, Strid T (2019). Diverse motif ensembles specify non-redundant DNA binding activities of AP-1 family members in macrophages. Nat Commun.

[41] He H, Song D, Sinha I, Hessling B, Li X, Haldosen L-A (2019). Endogenous interaction profiling identifies DDX5 as an oncogenic coactivator of transcription factor Fra-1. Oncogene.

[42] Verfaillie A, Imrichova H, Atak ZK, Dewaele M, Rambow F, Hulselmans G (2015). Decoding the regulatory landscape of melanoma reveals TEADS as regulators of the invasive cell state. Nat Commun.

[43] Zanconato F, Forcato M, Battilana G, Azzolin L, Quaranta E, Bodega B (2015). Genome-wide association between YAP/TAZ/TEAD and AP-1 at enhancers drives oncogenic growth. Nat Cell Biol.

[44] Liu X, Li H, Rajurkar M, Li Q, Cotton JL, Ou J (2016). Tead and AP1 coordinate transcription and motility. Cell Rep.

[45] Ndlovu ‘Matladi N, Van Lint C, Van Wesemael K, Callebert P, Chalbos D, Haegeman G (2009). Hyperactivated NF-{kappa}B and AP-1 transcription factors promote highly accessible chromatin and constitutive transcription across the interleukin-6 gene promoter in metastatic breast cancer cells. Mol Cell Biol.

[46] Crish JF, Eckert RL (2008). Synergistic activation of human involucrin gene expression by Fra-1 and p300–evidence for the presence of a multiprotein complex. J Invest Dermatol.

[47] Moquet-Torcy G, Tolza C, Piechaczyk M, Jariel-Encontre I (2014). Transcriptional complexity and roles of Fra-1/AP-1 at the uPA/Plau locus in aggressive breast cancer. Nucleic Acids Res.

[48] Tolza C, Bejjani F, Evanno E, Mahfoud S, Moquet-Torcy G, Gostan T (2019). AP-1 signaling by Fra-1 directly regulates HMGA1 oncogene transcription in triple-negative breast cancers. Mol Cancer Res MCR.

[49] Khoshakhlagh M, Soleimani A, Binabaj MM, Avan A, Ferns GA, Khazaei M (2019). Therapeutic potential of pharmacological TGF-β signaling pathway inhibitors in the pathogenesis of breast cancer. Biochem Pharmacol.

[50] Xiao C, Fan T, Tian H, Zheng Y, Zhou Z, Li S (2021). H3K36 trimethylation-mediated biological functions in cancer. Clin Epigenet.

[51] Harlen KM, Churchman LS (2017). The code and beyond: transcription regulation by the RNA polymerase II carboxy-terminal domain. Nat Rev Mol Cell Biol.

[52] Davies JOJ, Telenius JM, McGowan SJ, Roberts NA, Taylor S, Higgs DR (2016). Multiplexed analysis of chromosome conformation at vastly improved sensitivity. Nat Methods.

[53] Geeven G, Teunissen H, de Laat W, de Wit E (2018). peakC: a flexible, non-parametric peak calling package for 4C and capture-C data. Nucleic Acids Res.

[54] Andersson R, Gebhard C, Miguel-Escalada I, Hoof I, Bornholdt J, Boyd M (2014). An atlas of active enhancers across human cell types and tissues. Nature.

[55] Rao SSP, Huntley MH, Durand NC, Stamenova EK, Bochkov ID, Robinson JT (2014). A 3D map of the human genome at kilobase resolution reveals principles of chromatin looping. Cell.

[56] Bompadre O, Andrey G (2019). Chromatin topology in development and disease. Curr Opin Genet Dev.

[57] Luppino JM, Joyce EF (2020). Single cell analysis pushes the boundaries of TAD formation and function. Curr Opin Genet Dev.

[58] Dao LTM, Galindo-Albarrán AO, Castro-Mondragon JA, Andrieu-Soler C, Medina-Rivera A, Souaid C (2017). Genome-wide characterization of mammalian promoters with distal enhancer functions. Nat Genet.

[59] Syed KM, Hon C-C (2021). Heterogeneity among enhancer RNAs: origins, consequences and perspectives. Essays Biochem.

[60] Sartorelli V, Lauberth SM (2020). Enhancer RNAs are an important regulatory layer of the epigenome. Nat Struct Mol Biol.

[61] Arnold PR, Wells AD, Li XC (2019). Diversity and emerging roles of enhancer RNA in regulation of gene expression and cell fate. Front Cell Dev Biol.

[62] Franco HL, Nagari A, Malladi VS, Li W, Xi Y, Richardson D (2018). Enhancer transcription reveals subtype-specific gene expression programs controlling breast cancer pathogenesis. Genome Res.

[63] Rodríguez-Gil A, Riedlinger T, Ritter O, Saul VV, Schmitz ML (2018). Formaldehyde-assisted isolation of regulatory elements to measure chromatin accessibility in mammalian cells. J Vis Exp JoVE.

[64] Raisner R, Kharbanda S, Jin L, Jeng E, Chan E, Merchant M (2018). Enhancer activity requires CBP/P300 bromodomain-dependent histone H3K27 acetylation. Cell Rep.

[65] Narita T, Ito S, Higashijima Y, Chu WK, Neumann K, Walter J (2021). Enhancers are activated by p300/CBP activity-dependent PIC assembly, RNAPII recruitment, and pause release. Mol Cell.

[66] Breen ME, Mapp AK (2018). Modulating the masters: chemical tools to dissect CBP and p300 function. Curr Opin Chem Biol.

[67] Voss AK, Thomas T (2018). Histone lysine and genomic targets of histone acetyltransferases in mammals. BioEssays News Rev Mol Cell Dev Biol.

[68] Weinert BT, Narita T, Satpathy S, Srinivasan B, Hansen BK, Schölz C (2018). Time-resolved analysis reveals rapid dynamics and broad scope of the CBP/p300 acetylome. Cell.

[69] Matthews CP, Colburn NH, Young MR (2007). AP-1 a target for cancer prevention. Curr Cancer Drug Targets.

[70] Shetty A, Bhosale SD, Tripathi SK, Buchacher T, Biradar R, Rasool O (2021). Interactome networks of FOSL1 and FOSL2 in human Th17 cells. ACS Omega.

[71] Stinson S, Lackner MR, Adai AT, Yu N, Kim H-J, O’Brien C (2011). miR-221/222 targeting of trichorhinophalangeal 1 (TRPS1) promotes epithelial-to-mesenchymal transition in breast cancer. Sci Signal..

[72] Wu J, Sun Y, Zhang P-Y, Qian M, Zhang H, Chen X (2016). The Fra-1-miR-134-SDS22 feedback loop amplifies ERK/JNK signaling and reduces chemosensitivity in ovarian cancer cells. Cell Death Dis.

[73] Segert JA, Gisselbrecht SS, Bulyk ML (2021). Transcriptional silencers: driving gene expression with the brakes on. Trends Genet TIG.

[74] Bose DA, Donahue G, Reinberg D, Shiekhattar R, Bonasio R, Berger SL (2017). RNA binding to CBP stimulates histone acetylation and transcription. Cell.

[75] Dancy BM, Cole PA (2015). Protein lysine acetylation by p300/CBP. Chem Rev.

[76] Ray-Jones H, Spivakov M (2021). Transcriptional enhancers and their communication with gene promoters. Cell Mol Life Sci CMLS.

[77] Sharp PA, Chakraborty AK, Henninger JE, Young RA (2022). RNA in formation and regulation of transcriptional condensates. RNA N Y N.

[78] Wagh K, Garcia DA, Upadhyaya A (2021). Phase separation in transcription factor dynamics and chromatin organization. Curr Opin Struct Biol.

[79] Henninger JE, Oksuz O, Shrinivas K, Sagi I, LeRoy G, Zheng MM (2021). RNA-mediated feedback control of transcriptional condensates. Cell.

[80] Karr JP, Ferrie JJ, Tjian R, Darzacq X (2022). The transcription factor activity gradient (TAG) model: contemplating a contact-independent mechanism for enhancer-promoter communication. Genes Dev.

[81] Kovary K, Bravo R (1991). Expression of different Jun and Fos proteins during the G0-to-G1 transition in mouse fibroblasts: in vitro and in vivo associations. Mol Cell Biol.

[82] Bakiri L, Matsuo K, Wisniewska M, Wagner EF, Yaniv M (2002). Promoter specificity and biological activity of tethered AP-1 dimers. Mol Cell Biol.

[83] Buenrostro JD, Giresi PG, Zaba LC, Chang HY, Greenleaf WJ (2013). Transposition of native chromatin for fast and sensitive epigenomic profiling of open chromatin, DNA-binding proteins and nucleosome position. Nat Methods.

[84] Fenouil R, Descostes N, Spinelli L, Koch F, Maqbool MA, Benoukraf T (2016). Pasha: a versatile R package for piling chromatin HTS data. Bioinforma Oxf Engl.

