# Supplementary material for: Multiple Fra-1-bound enhancers showing different molecular and functional features can cooperate to repress gene transcription
Source: Cell Biosci. 2023 Jul 18;13:129. doi: 10.1186/s13578-023-01077-5 (PMC10354941; doi:10.1186/s13578-023-01077-5)
Supplement: Supplementary file 1 — Additional file 1: Data S1. Transcriptional repression of TGFB2 by Fra-1. The experiments presented in (A), (B) and (C) are the same as those presented in Figure 1B, -C and -D, except that other amplicons were used. The sequences of the oligonucleotides used in RT-qPCR assays are given in Additional file 7: Table S1B. (D) RPS26 gene and ChIP-qPCR. The upper panel shows the amplicon positions used in ChIP-qPCR experiments, indicated in kb from the TSS. The middle panel shows ChIP-qPCR analysis of total Pol II on the RPS26 gene (n=4) and the lower panel shows ChIP-qPCR analysis of Pol II-PSer5 on the RPS26 gene (n=4). All values were normalized to that of amplicon − 0.8 kb under control condition arbitrarily set to 1. The arrows indicate the RPS26 TSS. All experiments were carried out using MDA-MB-231 cells transfected with either siFra-1 (green boxes) or siCTL (violet boxes) for 72 hours. (E) Expression levels of the genes located in the TGFB2 TAD upon Fra-1 down-regulation. The transcriptome regulated by Fra-1 was formerly identified using Affymetrix GeneChip Human Gene 2.0 ST arrays [36]. Gene expression ratios in siFra-1 versus siCTL conditions showed that the mRNA steady-state levels of RRP15, LYPLAL1 and LINC02869 were not affected upon Fra-1 down-regulation. Expression of LYPLAL1-DT, LYPLAL1-AS1, ZC3H11B and LINC01710 could not be analyzed (NA), as the corresponding probes were absent from the Affymetrix arrays used. [file 13578_2023_1077_MOESM1_ESM.pdf]

Additional Data S1

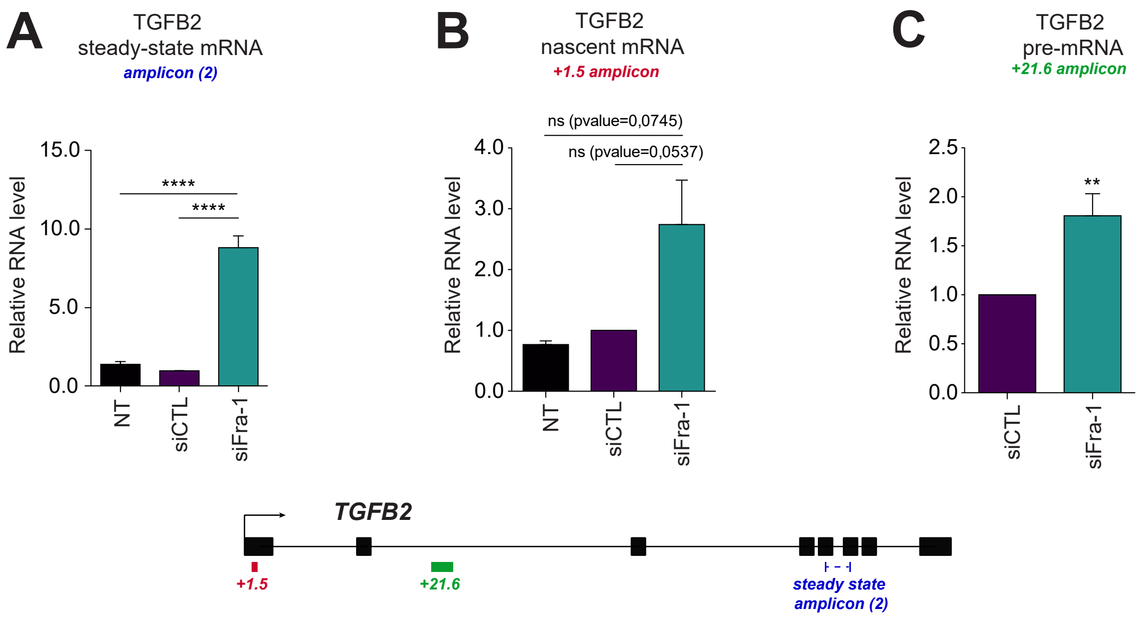

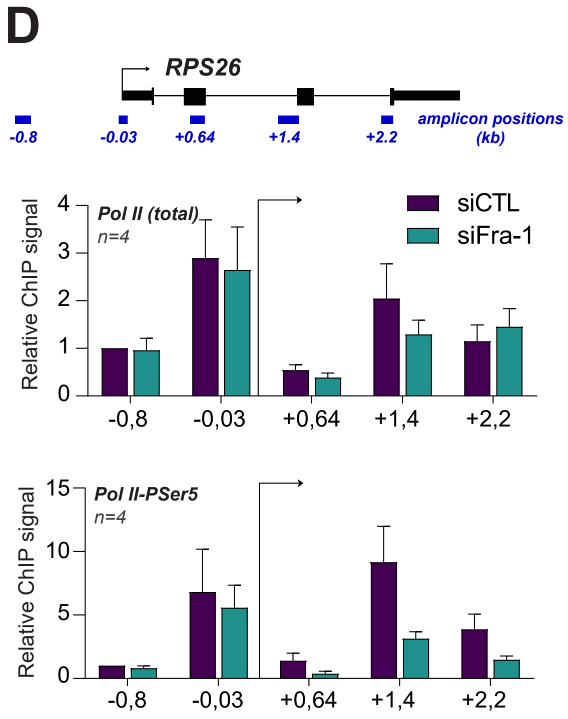

E

Gene	siFra-1/siCTL
RRP15	1
LYPLAL1	1
LYPLAL1-DT	NA
LYPLAL1-AS1	NA
ZC3H11B	NA
LINC02869 / C1orf143 / Lnc-TGFB2-1	1
LINC01710	NA

Additional Data S1: Transcriptional repression of TGFB2 by Fra-1. The experiments presented in (A), (B) and (C) are the same as those presented in Figure 1B, -C and -D, except that other amplicons were used. The sequences of the oligonucleotides used in RT-qPCR assays are given in Additional file 7: Table S1B. (D) RPS26 gene and ChIP-qPCR. The upper panel shows the amplicon positions used in ChIP-qPCR experiments, indicated in kb from the TSS. The middle panel shows ChIP-qPCR analysis of total Pol II on the RPS26 gene (n=4) and the lower panel shows ChIP-qPCR analysis of Pol II-PSer5 on the RPS26 gene (n=4). All values were normalized to that of amplicon − 0.8 kb under control condition arbitrarily set to 1. The arrows indicate the RPS26 TSS. All experiments were carried out using MDA-MB-231 cells transfected with either siFra-1 (green boxes) or siCTL (violet boxes) for 72 hours. (E) Expression levels of the genes located in the TGFB2 TAD upon Fra-1 down-regulation. The transcriptome regulated by Fra-1 was formerly identified using Affymetrix GeneChip Human Gene 2.0 ST arrays [36]. Gene expression ratios in siFra-1 versus siCTL conditions showed that the mRNA steady-state levels of RRP15, LYPLAL1 and LINC02869 were not affected upon Fra-1 down-regulation. Expression of LYPLAL1-DT, LYPLAL1-AS1, ZC3H11B and LINC01710 could not be analyzed (NA), as the corresponding probes were absent from the Affymetrix arrays used.
