# Supplementary material for: Multiple Fra-1-bound enhancers showing different molecular and functional features can cooperate to repress gene transcription
Source: Cell Biosci. 2023 Jul 18;13:129. doi: 10.1186/s13578-023-01077-5 (PMC10354941; doi:10.1186/s13578-023-01077-5)
Supplement: Supplementary file 2 — Additional file 2: Data S2. Positions of AP-1 motifs under the Fra-1 ChIP-seq peaks at the Fra-1-bound TGFB2 enhancers. The positions of the Fra-1-bound enhancers are given with respect to the TGFB2 TSS. AP-1 motif positions are given both with respect to the TGFFB2 TSS and in the reference genome Hg19. The sequences of the AP-1- and AP-1-related motifs are indicated in black and blue, respectively. [file 13578_2023_1077_MOESM2_ESM.pdf]

Additional Data S2

Fra-1 peak	AP-1 motif	Distance to TGFB2 TSS	Genomic location
+32	TGAGTCA	+32.16	chr1:218550784-218550791
+115	TGACTCA	+114.83	chr1:218633454-218633461
+118	TGACTCA	+118.40	chr1:218637030-218637037
+135	TGACTCA	+135.56	chr1:218654183-218654190
+151	TGAATCA	+151.45	chr1:218670073-218670080
+241	TGAGTCA	+240.38	chr1:218759008-218759015
+314	TGACTCA	+313.80	chr1:218832431-218832438
+315-1	TGACTCA	+315.57	chr1:218834198-218834205
+315-2	TGAGTCA	+315.63	chr1:218834262-218834269
+360	TGACTGA	+360.76	chr1:218879383-218879390
+744	TGACTCA	+743.87	chr1:219262496-219262503
+980	TGACTCA	+979.95	chr1:219498576-219498583
+1426-1	TGACTCA	+1425.89	chr1:219944522-219944529
+1426-2	TGACTCA	+1425.98	chr1:219944611-219944618

Additional Data S2. Positions of AP-1 motifs under the Fra-1 ChIP-seq peaks at the Fra-1-bound TGFB2 enhancers. The positions of the Fra-1-bound enhancers are given with respect to the TGFB2 TSS. AP-1 motif positions are given both with respect to the TGFB2 TSS and in the reference genome Hg19. The sequences of the AP-1- and AP-1-related motifs are indicated in black and blue, respectively.
