# Supplementary material for: Multiple Fra-1-bound enhancers showing different molecular and functional features can cooperate to repress gene transcription
Source: Cell Biosci. 2023 Jul 18;13:129. doi: 10.1186/s13578-023-01077-5 (PMC10354941; doi:10.1186/s13578-023-01077-5)
Supplement: Supplementary file 3 — Additional file 3: Data S3. Molecular features of the TGFB2 PIRs. ChIP-seq data for H3K4me3, H3K27ac, H3K4me1, Pol II, CTCF, p300/CBP, Fra-1, as well as ATAC-seq data, were used to, not only classify the 15 TGFB2 PIRs defined by NG Capture-C in MDA-MB-231 cells, but also show their molecular heterogeneity. The first category contains the PIRs harboring only a Fra-1-bound candidate active enhancer (PIRs +32, +136, + 151, +240, +360 and +980). These are all characterized by overlapping Fra-1- and ATAC-seq signals and absence of CTCF and H3K4me3 signals. However, they show some heterogeneity in H3K4me1, H3K27ac, Pol II, and p300/CBP signal intensities. The second category contains PIRs marked by CTCF but not by Fra-1 (PIR -46, +1041, +1222 and +1316). At variance with Fra-1-bound active enhancers, CTCF-bound elements are not associated with ATAC-seq signals. The third category corresponds to a PIR (PIR +836) that is not bound by Fra-1 but bears an active gene promoter (open chromatin configuration in ATAC-seq experiments and strongly marked by H3K4me1, H3K4me3 and H3K27ac) with possible enhancer function for TGFB2 (Epromoter). The fourth category contains PIRs (PIR +116 and +314) that carry two Fra-1-bound active enhancers specified by overlapping Fra-1- and ATAC-seq signals. Like in the first category of PIRs, the candidate enhancers are not marked by CTCF and H3K4me3 and show some heterogeneity in H3K4me1, H3K27ac, Pol II, and p300/CBP signals. Finally, the fifth category (PIRs +729 and +1449) contains PIRs bearing one Fra-1-bound active enhancer, as well as at least one CTCF-binding elements. [file 13578_2023_1077_MOESM3_ESM.pdf]

Additional Data S3

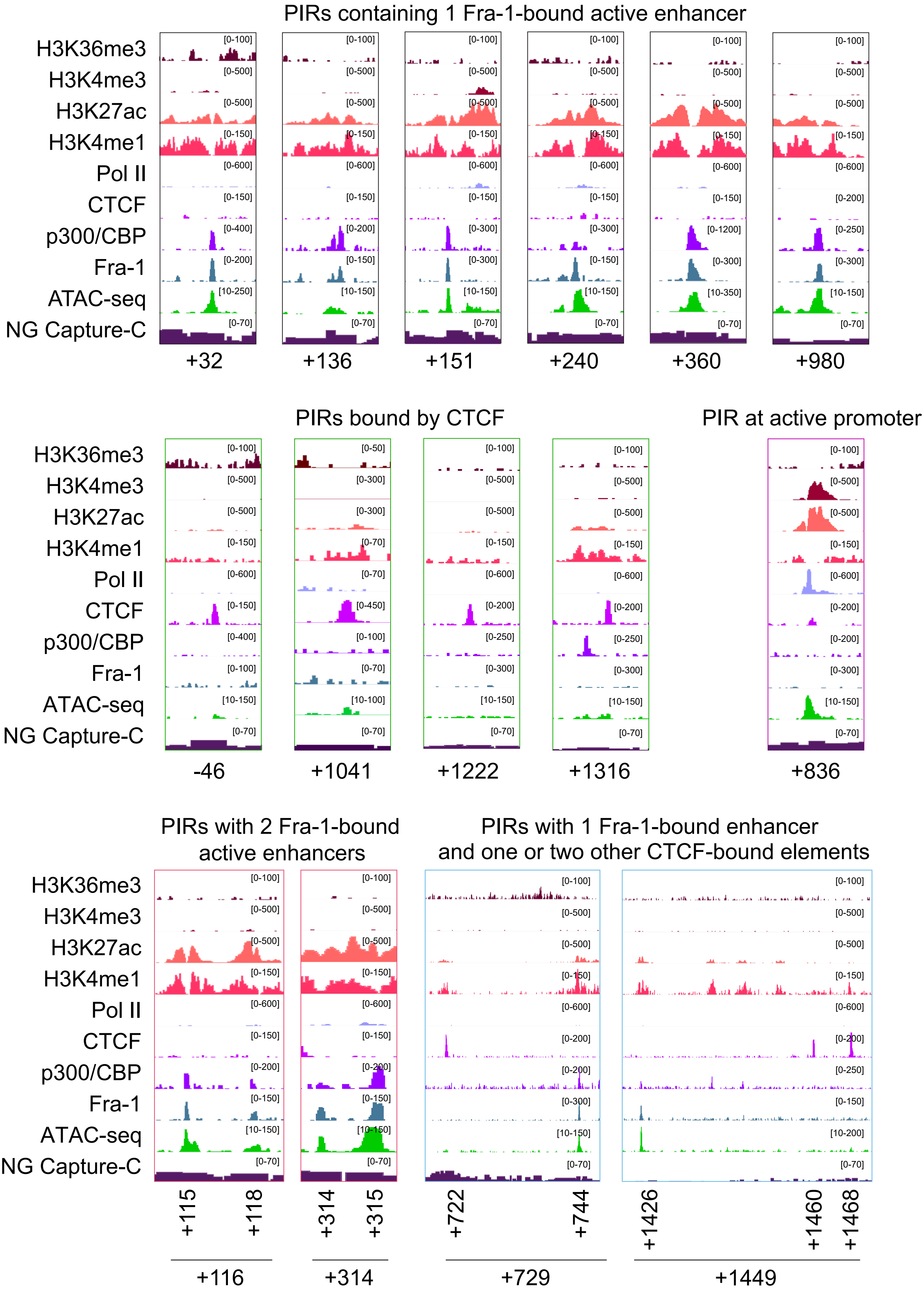

Additional Data S3: Molecular features of the TGFB2 PIRs. ChIP-seq data for H3K4me3, H3K27ac, H3K4me1, Pol II, CTCF, p300/CBP, Fra-1, as well as ATAC-seq data, were used to, not only classify the 15 TGFB2 PIRs defined by NG Capture-C in MDA-MB-231 cells, but also show their molecular heterogeneity. The first category contains the PIRs harboring only a Fra-1-bound candidate active enhancer (PIRs +32, +136, + 151, +240, +360 and +980). These are all characterized by overlapping Fra-1- and ATAC-seq signals and absence of CTCF and H3K4me3 signals. However, they show some heterogeneity in H3K4me1, H3K27ac, Pol II, and p300/CBP signal intensities. The second category contains PIRs marked by CTCF but not by Fra-1 (PIR -46, +1041, +1222 and +1316). At variance with Fra-1-bound active enhancers, CTCF-bound elements are not associated with ATAC-seq signals. The third category corresponds to a PIR (PIR +836) that is not bound by Fra-1 but bears an active gene promoter (open chromatin configuration in ATAC-seq experiments and strongly marked by H3K4me1, H3K4me3 and H3K27ac) with possible enhancer function for TGFB2 (Epromoter). The fourth category contains PIRs (PIR +116 and +314) that carry two Fra-1-bound active enhancers specified by overlapping Fra-1- and ATAC-seq signals. Like in the first category of PIRs, the candidate enhancers are not marked by CTCF and H3K4me3 and show some heterogeneity in H3K4me1, H3K27ac, Pol II, and p300/CBP signals. Finally, the fifth category (PIRs +729 and +1449) contains PIRs bearing one Fra-1-bound active enhancer, as well as at least one CTCF-binding elements.
