# Supplementary material for: Multiple Fra-1-bound enhancers showing different molecular and functional features can cooperate to repress gene transcription
Source: Cell Biosci. 2023 Jul 18;13:129. doi: 10.1186/s13578-023-01077-5 (PMC10354941; doi:10.1186/s13578-023-01077-5)
Supplement: Supplementary file 4 — Additional file 4: Data S4. Bidirectional transcription at the TGFB2 locus in MDA-MB-231 cells. Nascent RNA production was assessed by others in MDA-MB-231 cells using Gro-seq [62]. The publicly available Gro-seq data (upper panel) were aligned along with the TGFB2 locus NG Capture-C data (lower panel), as well as Fra-1 ChIP-seq data obtained in Bejjani et al. [36]. Purple signals indicate sense (+) transcription with respect to TGFB2 gene transcription and orange ones antisense (-) transcription. [file 13578_2023_1077_MOESM4_ESM.pdf]

# Additional Data S4

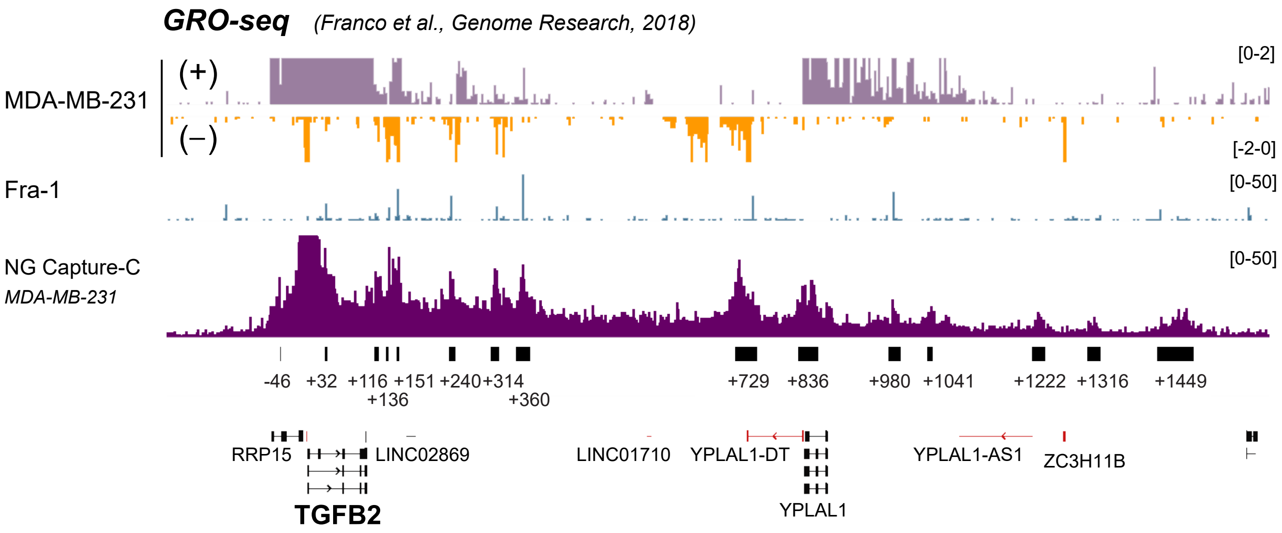


**Additional Data S4: Bidirectional transcription at the TGFB2 locus in MDA-MB-231 cells.** Nascent RNA production was assessed by others in MDA-MB-231 cells using Gro-seq [62]. The publicly available Gro-seq data (upper panel) were aligned along with the TGFB2 locus NG Capture-C data (lower panel), as well as Fra-1 ChIP-seq data obtained in Bejjani et al. [36]. Purple signals indicate sense (+) transcription with respect to TGFB2 gene transcription and orange ones antisense (-) transcription.
