# Supplementary material for: Multiple Fra-1-bound enhancers showing different molecular and functional features can cooperate to repress gene transcription
Source: Cell Biosci. 2023 Jul 18;13:129. doi: 10.1186/s13578-023-01077-5 (PMC10354941; doi:10.1186/s13578-023-01077-5)
Supplement: Supplementary file 5 — Additional file 5: Data S5. assay of eRNAs at the +32 Fra-1-bound PIR. (A) Molecular features of intron 2 from position 218,536,777 to 218,577,587. The Fra-1-binding site located at +32 kb resides in a region of open chromatin (ATAC-seq signal) also recruiting p300/CBP and marked by H3K4me1 and H3K27ac but not by CTCF. In contrast, there is no sign of open chromatin at positions +21.6 kb and +55.8 kb, as well as of marks specifying active enhancers. The amplicons used to assay eRNA versus TGFB2 pre-mRNA at position +32 kb are indicated by black boxes. (B) RT-qPCR assay of eRNA versus TGFB2 pre-mRNA at position +32 kb in MDA-MB-231 cells. RNA levels at positions +21.6-, 32- and 55.8 kb from the TGFB2 TSS were quantified by RT-qPCR. RNA amplified at position +21.6- and +55.8 kb correspond to pre-mRNA, whereas RNA amplified at position +32 kb corresponds to pre-mRNA+eRNA. The data are the results of 6 independent experiments. Relative RNA abundances at the 3 positions were calculated by the ∆∆CT method, amplicon +32 being taken as the reference. Primer sequences for qPCR amplification are given in Additional file 7: Table S1B. [file 13578_2023_1077_MOESM5_ESM.pdf]

Additional Data S5

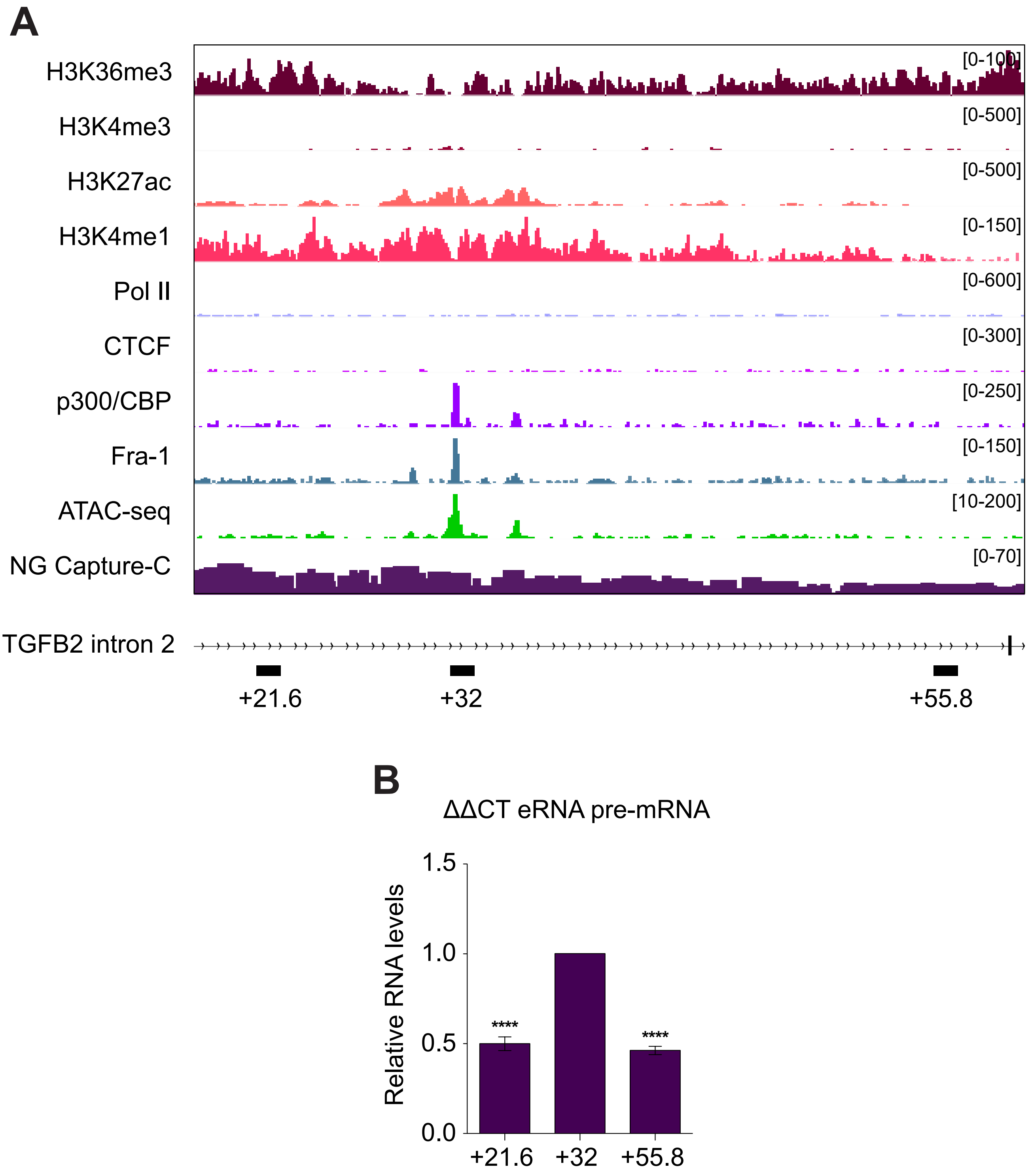

Additional Data S5. Assay of eRNAs at the +32 Fra-1-bound PIR. (A) Molecular features of intron 2 from position 218,536,777 to 218,577,587. The Fra-1-binding site located at +32 kb resides in a region of open chromatin (ATAC-seq signal) also recruiting p300/CBP and marked by H3K4me1 and H3K27ac but not by CTCF. In contrast, there is no sign of open chromatin at positions +21.6 kb and +55.8 kb, as well as of marks specifying active enhancers. The amplicons used to assay eRNA versus TGFB2 pre-mRNA at position +32 kb are indicated by black boxes. (B) RT-qPCR assay of eRNA versus TGFB2 pre-mRNA at position +32 kb in MDA-MB-231 cells. RNA levels at positions +21.6-, 32- and 55.8 kb from the TGFB2 TSS were quantified by RT-qPCR. RNA amplified at position +21.6- and +55.8 kb correspond to pre-mRNA, whereas RNA amplified at position +32 kb corresponds to pre-mRNA+eRNA. The data are the results of 6 independent experiments. Relative RNA abundances at the 3 positions were calculated by the ΔΔCT method, amplicon +32 being taken as the reference. Primer sequences for qPCR amplification are given in Additional Table S1B.
