# Supplementary material for: Multiple Fra-1-bound enhancers showing different molecular and functional features can cooperate to repress gene transcription
Source: Cell Biosci. 2023 Jul 18;13:129. doi: 10.1186/s13578-023-01077-5 (PMC10354941; doi:10.1186/s13578-023-01077-5)
Supplement: Supplementary file 6 — Additional file 6: Data S6. ATAC-seq signal ratios obtained in the absence and presence of Fra-1 at the Fra-1-bound TGFB2 enhancers. Three independent ATAC-seq experiments were carried out. (A) The ratios of ATAC-seq signal intensities in siFra-1 versus siCTL conditions are given for the 12 Fra-1-bound enhancers, as well as for the TGFB2 promoter. The start and end positions of the ATAC-seq peaks are given as well as the peak positions relative to the TGFB2 TSS. (B) The ratios of ATAC-seq signal intensities in siFra-1 versus siCTL conditions are given for two peaks (peak A on chromosome 16 and peak B on chromosome 15) for which the ATAC-seq signals were found increased or decreased (FC≥±1.5), respectively. The start and end position of peaks are given in the table. (C) The ratios of p300/CBP signal intensities in siFra-1 versus siCTL conditions at the Fra-1-bound TGFB2 enhancers showing a FC ≥ ±1.5 are indicated. ChIP-seq data obtained in MDA-MB-231 cells were previously presented in Bejjani et al. [36]. The peak positions relative to the TGFB2 TSS, as well as the start and end positions of the p300/CBP peaks are given. [file 13578_2023_1077_MOESM6_ESM.pdf]

Additional Data S6

A

chr	start	end	ATAC-seq FC siFra1/siCTL	peak position
chr1	218550691	218550961	1.23	32
chr1	218633391	218633561	1.32	115
chr1	218669941	218670261	1.45	118
chr1	218669941	218670261	1.49	136
chr1	218669941	218670261	1.45	151
chr1	218758891	218759161	1.49	240
chr1	218832341	218832561	-1.44	314
chr1	218834041	218834361	-1.09	315
chr1	218879241	218879861	1.22	360
chr1	219262341	219262611	1.06	744
chr1	219498391	219498711	-1.35	980
chr1	219944441	219944661	-1.30	1426
chr1	218518352	218518851	1.43	promoter

B

chr	start	end	ATAC-seq FC siFra1/siCTL	Peak
chr16	48161041	48161311	1.83	A
chr12	11465341	114165861	-2.288	B

C

chr	start	end	p300/CBP FC (siFra-1/siCTL)	peak position
chr1	218633401	218633551	2.98	115
chr1	218654101	218654301	4.59	136
chr1	218669951	218670251	2.12	151
chr1	218879251	218879851	1.81	360

Additional Data S6. ATAC-seq signal ratios obtained in the absence and presence of Fra-1 at the Fra-1-bound TGFB2 enhancers. Three independent ATAC-seq experiments were carried out. (A) The ratios of ATAC-seq signal intensities in siFra-1 versus siCTL conditions are given for the 12 Fra-1-bound enhancers, as well as for the TGFB2 promoter. The start and end positions of the ATAC-seq peaks are given as well as the peak positions relative to the TGFB2 TSS. (B) The ratios of ATAC-seq signal intensities in siFra-1 versus siCTL conditions are given for two peaks (peak A on chromosome 16 and peak B on chromosome 15) for which the ATAC-seq signals were found increased or decreased (FC≥±1.5), respectively. The start and end position of peaks are given in the table. (C) The ratios of p300/CBP signal intensities in siFra-1 versus siCTL conditions at the Fra-1-bound TGFB2 enhancers showing a FC ≥ ±1.5 are indicated. ChIP-seq data obtained in MDA-MB-231 cells were previously presented in Bejjani et al. [36]. The peak positions relative to the TGFB2 TSS, as well as the start and end positions of the p300/CBP peaks are given.
