# Supplementary material for: Multiple Fra-1-bound enhancers showing different molecular and functional features can cooperate to repress gene transcription
Source: Cell Biosci. 2023 Jul 18;13:129. doi: 10.1186/s13578-023-01077-5 (PMC10354941; doi:10.1186/s13578-023-01077-5)
Supplement: Supplementary file 7 — Additional file 7. Additional Tables. [file 13578_2023_1077_MOESM7_ESM.pdf]

Additional Tables

Additional Table S1A: Sequences or references of the siRNA used in this study

siRNA	target	Strand	Sequence (5' - 3')
Pool siFra-1	siFra-1-A	Forward	CCAAAGCUGCCCACUGUUU-dTdT
	siFra-1-A	Reverse	AAACAGUGGGCAGCUUUGG-dTdT
	siFra-1-B	Forward	GACAGUAUCCCACAUCCAA-dTdT
	siFra-1-B	Reverse	UUGGAUGUGGGAUACUGUC-dTdT
	siFra-1-C	Forward	CUGACUGCCACUCAUGGUG-dTdT
	siFra-1-C	Reverse	CACCAUGAGUGGCAGUCAG-dTdT
siCTL	Silencer® Negative Control N°1 siRNA. Ambion # AM4611		
sip300 Thermofisher	Ep300 silencer@select predesigned siRNA ref 4392420. ID S4695		
siCBP Thermofisher	CBP silencer@select predesigned siRNA ref 4390824. ID S3495		

Additional Table S1B: sequences of the primers used for mRNA, nascent mRNA or pre-mRNA quantification by RT-qPCR

mRNA amplicons	Strand	Sequence
RPS26	Forward	CTGCACTAACTGTGCCCGATGCGTG
	Reverse	GACGCTCGCTTCAGAAATGTCCCTG
TGFB2-1	Forward	TTGCAGAACCCAAAAGCCAGA
	Reverse	TGTCGATGTAGCGCTGGGTTG
TGFB2-2	Forward	AAGCTTACACTGTCCCTGCT
	Reverse	ACTGGTATATGTGGAGGTGCC

Run-On amplicons	Strand	Sequence
GAPDH +2.4	Forward	AATCCCATCACCATCTTCCAG
	Reverse	GAGCCACACCATCCTAGTTG
TGFB2 +3.7	Forward	AGAGCTGAGATGATCCAACCC
	Reverse	GGCTGGGGTGAAATGGAAAGG
TGFB2 +1.05	Forward	CACCCGAGACTGACACACTGA
	Reverse	AACCCTGACTTTGGCGAGTAA

pre-mRNA amplicons	Strand	Sequence
TGFB2 +21.6	Forward<br>Reverse	GTCACCAGGCTAGAAGGCAGT<br>AAATTAGCTGGGAGTGGCGGC
TGFB2 +32	Forward<br>Reverse	ACCCCAGCATCCATCTTCCTG<br>GTGGTGGCAAATGGGAAGCC
TGFB2 +55.8	Forward<br>Reverse	GTGACCCAGAGCAGATGACAG<br>TTCCCTGCCTGTACTCCTGCT

Additional Table S1C: sequences and positions of the primers used for eRNA amplifications

PIR	Fra-1 peak position	Strand	Sequence	amplicon size (nt)	nt from Fra-1 peak
**+32**	+32	Forward	ACCCCAGCATCCATCTTCCTG	70	+400
		Reverse	GTGGTGGCAAATGGGAAGCC		
**+116**	+115	Forward	TTCTGCACACTCTCCTCCCTG	72	+110
		Reverse	TTCAGGCAGGGGACAAAGAAG		
	+118	Forward	CCTCTTTCTTGGTCCCCTCTC	177	+128
		Reverse	GAGTGGTGTTTTTCCCGCCCT		
**+136**	+136	Forward	AAATGGAGTGCAAGACAGGCT	139	+213
		Reverse	ATGCCAGAGATAGCCATTCCA		
**+151**	+151	Forward	GAGCCTGGAGTGTTGCAGTAT	118	+160
		Reverse	GCGCCCTATAAATCAGAGGAC		
**+240**	+240	Forward	CCCCAACTAAACGGCTACCTC	118	+91
		Reverse	CCACCCTCTGCTTCTATGACT		
**+314**	+314	Forward	AAAAGCTGACCCCACCTGTATC	211	-105
		Reverse	AGTTCCCAGACCCAATTCTTCC		
	+315	Forward	GGGGATTGCCATTGGATCTTT	67	+155
		Reverse	ACCAACTTGCAAGATGAAGGG		
**+360**	+360	Forward	ACACTCCTCCTCATCACTCCC	120	+291
		Reverse	GGGAGGCTGGATGACAGATAC		
**+729**	+744	Forward	AGAACTCAGCCAGTGCCCATG	119	-275
		Reverse	GTGCTTTGGCCCATGGTAGTG		
**+980**	+980	Forward	GTCTAAACCACCCCTTCCCTG	72	+146
		Reverse	GTCAAGAAAGGGACGGTGCCA		
**+1449**	+1426	Forward	AAGCTTAGTTGCCTAGCCTGG	98	+157
		Reverse	GAGGGAGTTGCATTGAATCAG		

Additional Table S1D: primers used for ChIP-qPCR and FAIRE-qPCR amplification

Primers for control regions in FAIRE-qPCR experiments

Amplicon	Coordinates	Strand	Sequence
A	chr16:48.161.051-48.161.301	Forward	CTGGGAGTCTGCTGCTGAGTT
		Reverse	AGGCAACGCTGTGGGTTTAGG
B	chr15:63.061.901-63.062.101	Forward	GTCTTCCTGTCCACATCCCCA
		Reverse	CAAACCTTTAGCCAGCACCT

Primers for *TGFB2* locus amplification (ChIP-qPCR and FAIRE-qPCR)

Primer location	Distance from TSS (Kb)	Strand	Sequence
**upstream**	-2.3	Forward	ATTTGAGAGGTGGAAAGGGGC
		Reverse	AGGAGGGTAAGACGGGAGGTA
	-1.0	Forward	AGGCCCCATACACAACTGAAG
		Reverse	AGCCATACTGACCAGACAGAT
**gene body**	-0.1	Forward	CACATTCCACCTCCTTCCTCC
		Reverse	GCCTTCAACAAAGTGACGTGC
	+1.0	Forward	CACCCGAGACTGACACACTGA
		Reverse	AACCCTGACTTTGGCGAGTAA
	+2.9	Forward	TGCTCATTTCTCTCCTCCCCT
		Reverse	CCACCCTGCAACTAGAACCTT
	+3.7	Forward	AGAGCTGAGATGATCCAACCC
		Reverse	GGCTGGGGTGAAATGGAAAGG
	+11.1	Forward	TTTGTAGAGCTGGGGTCTTGT
		Reverse	GAAGGCAGAGGCAGGAGGATT
	+32.1	Forward	GAACCCAGTGCTTTACGTGGC
		Reverse	GATTTGGGGGTGGAGCTGGTG
	+67.2	Forward	ACCTTCCTCGTGTTTCAGTGT
		Reverse	ACGCCCATACCATTGCATACT
	+84.7	Forward	AGAGTGGAAGTGCTTGGAGAG
		Reverse	GGCCAGAGTAACCAGCATTCT

**downstream enhancers**	+115	Forward	ACAGCAGGGTGTTCAGCTTTA
		Reverse	TGCTCCAAATAACTTGCCCCT
	+118	Forward	TCCTGGGACCAACACCTAACC
		Reverse	GGCCCAGTGAGTCATGCAGAA
	+136	Forward	CAGACTGATGGGGAGATATTCTTC
		Reverse	CCTGTCTTGCACTCCATTTTACAA
	+151	Forward	CTGGGCATTTAGGGGAGGGTG
		Reverse	ATACTGCAACACTCCAGGCTC
	+240	Forward	ACCTTGGCAGAGAGTAAACCT
		Reverse	CCGGCTGTGTGACGCATAACA
	+314	Forward	GAGCAGGACCACAGAACCACA
		Reverse	CCCAGTCAGCTAGTCATTCGG
	+315	Forward	AGGGTCCATAAACTAGAGTGT
		Reverse	CTCAGCGTCAATGCATTCCCA
	+360	Forward	GTGGTGAAGTTGCTCCGAACC
		Reverse	TGCAAAGCTCTACCTGACTCC
	+744	Forward	CCAGGCAGTGCAGCTCAGTAA
		Reverse	TTGTGGAGGGGTGATGCAAGC
	+980	Forward	GAGCAGTGGGTCAGGCCAGAT
		Reverse	GGGGTGGGTATAGGGAGTGAG
	+1426	Forward	AACTCCAAACGCCCCTCCAAG
		Reverse	ACCCAGGAGACAAGCTACAAT
**negative control**	NC +481	Forward	TGCACACAACAAGGGTGGTCC
		Reverse	GCAAGAGAGGAGGAACACAGC

Primers for *RPS26* locus amplification (ChIP-qPCR)

Primer location	Distance from TSS (Kb)	Strand	Sequence
**upstream**	-0.8	Forward	CGTGTTTGTGTGTGTACTGGG
		Reverse	TGGCTCCGCTGTACTATGCTG
**Promoter**	-0.03	Forward	CAGCGTCTGGCAACTTTCACGCCTAGC
		Reverse	GCCGGATGGCGGAAGAAAATCGAGGTTATGTG
	+0.64	Forward	CTGCACTAACTGTGCCCGATGCGTG
		Reverse	GACGCTCGCTTCAGAAATGTCCCTG
**Gene body**	+1.4	Forward	TGGGCTGAACAGGTGCTTTGG
		Reverse	GGGAAGCACATAGGCTAAGGA
	+2.2	Forward	GTTCTTTGGGGGAAGGGAGTC
		Reverse	TTACATGGGCTTTGGTGGGGG
